# Genome-wide analysis identifies novel loci influencing plasma apolipoprotein E concentration and Alzheimer’s disease risk

**DOI:** 10.1038/s41380-023-02170-4

**Published:** 2023-09-05

**Authors:** M. Muaaz Aslam, Kang-Hsien Fan, Elizabeth Lawrence, Margaret Anne Bedison, Beth E. Snitz, Steven T. DeKosky, Oscar L. Lopez, Eleanor Feingold, M. Ilyas Kamboh

**Affiliations:** 1 Department of Human Genetics, School of Public Health, University of Pittsburgh, Pittsburgh, PA USA; 2 Department of Neurology, School of Medicine, University of Pittsburgh, Pittsburgh, PA USA; 3 McKnight Brain Institute and Department of Neurology, College of Medicine, University of Florida, Gainesville, FL USA

**Keywords:** Genetics, Predictive markers

## Abstract

The *APOE 2/3/4* polymorphism is the greatest genetic risk factor for Alzheimer’s disease (AD). This polymorphism is also associated with variation in plasma ApoE level; while APOE*4 lowers, APOE*2 increases ApoE level. Lower plasma ApoE level has also been suggested to be a risk factor for incident dementia. To our knowledge, no large genome-wide association study (GWAS) has been reported on plasma ApoE level. This study aimed to identify new genetic variants affecting plasma ApoE level as well as to test if baseline ApoE level is associated with cognitive function and incident dementia in a longitudinally followed cohort of the Ginkgo Evaluation of Memory (GEM) study. Baseline plasma ApoE concentration was measured in 3031 participants (95.4% European Americans (EAs)). GWAS analysis was performed on 2580 self-identified EAs where both genotype and plasma ApoE data were available. Lower ApoE concentration was associated with worse cognitive function, but not with incident dementia. As expected, the risk for AD increased from E2/2 through to E4/4 genotypes (*P* for trend = 4.8E-75). In addition to confirming the expected and opposite associations of APOE*2 (*P* = 4.73E-79) and APOE*4 (*P* = 8.73E-12) with ApoE level, GWAS analysis revealed nine additional independent signals in the *APOE* region, and together they explained about 22% of the variance in plasma ApoE level. We also identified seven new loci on chromosomes 1, 4, 5, 7, 11, 12 and 20 (*P* range = 5.49E-08 to 5.36E-10) that explained about 9% of the variance in ApoE level. Plasma ApoE level-associated independent variants, especially in the *APOE* region, were also associated with AD risk and amyloid deposition in the brain, indicating that genetically determined ApoE level variation may be a risk factor for developing AD. These results improve our understanding of the genetic determinants of plasma ApoE level and their potential value in affecting AD risk.

## Introduction

Apolipoprotein E (ApoE protein; *APOE* gene), a 299 amino acid long multifunctional glycoprotein (34-k Da), serves as the transporter of cholesterol and other lipids in the central nervous system (CNS) and in plasma via binding to cell surface ApoE receptors [1, 2]. While plasma ApoE is derived primarily from hepatocytes, in CNS it is mainly produced in astrocytes [3]. ApoE plays a pivotal role in the CNS by transporting cholesterol and phospholipids to neurons, which is crucial for neurodevelopment, neuronal repair, and neurotransmission. There is a common three-allele *APOE* polymorphism: APOE*2, APOE*3, and APOE*4, resulting in six genotypes (2/2,2/3,2/4,3/3,3/4,4/4), and it has a profound effect on determining interindividual variation in plasma cholesterol level and in determining Alzheimer’s disease (AD) risk [2–5].

AD is a gradually progressive, heterogenous, irreversible, and detrimental neurodegenerative disorder and the leading cause of dementia in the geriatric population accounting for almost 60–80% of all dementia cases [6]. The *APOE* polymorphism is the most studied, successfully replicated, and well-established risk factor for AD where APOE*2 is the protective allele and APOE*4 is the risk allele as compared to APOE*3 [7–10]. The effect of APOE*4 is dose-dependent; one and two copies of E*4 increase the AD risk by 3.5- and 14.5-fold, respectively [10]. APOE*4 is also associated with AD-related proteinopathies, including amyloid-β, tau, α-synuclein, and TDP-43 [11, 12]. APOE*4 may also have direct pathologic effects on neurons and the blood-brain barrier (BBB) function independent of its effects on amyloid and tau pathologies [12].

*APOE* polymorphism is also associated with variation in plasma ApoE concentration; APOE*4 is associated with lower and APOE*2 with higher level as compared to the common APOE*3 allele [13, 14]. Lower plasma ApoE level has also been reported to be a risk factor for incident dementia and AD, independent of the *APOE 2/3/4* polymorphism [14, 15]. Previously a small genome-wide association study (GWAS) was carried out on only 570 subjects with plasma ApoE level that identified only the known signal in the *APOE* region [16]. Here we performed the largest GWAS to identify novel genetic factors affecting plasma ApoE level as well as to test if baseline plasma ApoE level affects cognitive function and incident dementia in a longitudinal cohort of the Ginkgo Evaluation of Memory (GEM) study [17–19].

## Results

The details of study participants, plasma ApoE measurement, genotyping and imputation, statistical analyses, and functional annotations are given in Online Methods.

### Association of plasma ApoE concentration with incident dementia and cognition function

**Fig. 1:**
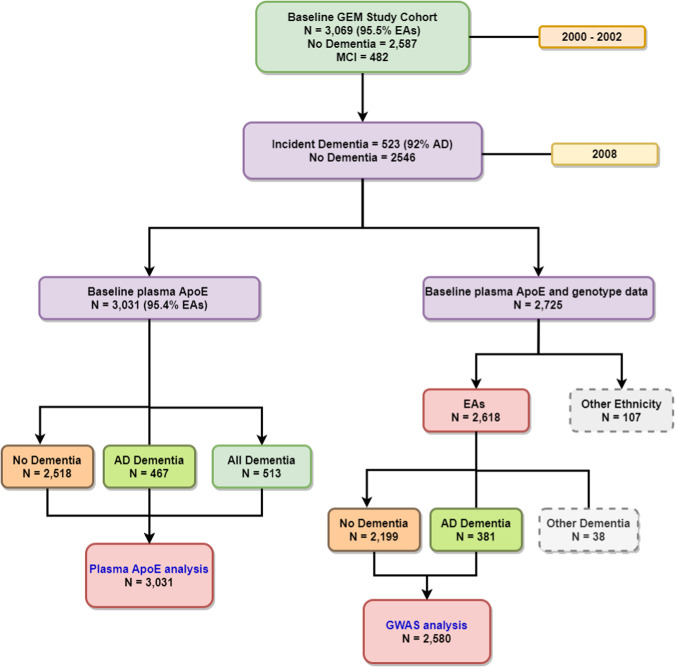
Flow chart of the GEM study subjects included in the plasma ApoE analysis in the total sample and plasma ApoE-genotype analysis in European Americans (EAs). MCI = Mild cognitive impairment. Boxes with broken lines indicate that they were not included in the analysis.

Whole plasma ApoE concentration at baseline was determined in 3031 participants, of which 2893 were European Americans (EAs), including 2412 who remained non-demented (ND) and 481 with incident dementia (91.4% AD dementia) (Fig. 1). Plasma ApoE level followed a symmetric distribution, ranging from 0.50 to 15.70 mg/dl with a mean value of 4.1 ± 1.25 mg/dL in the total sample and 4.09 ± 1.25 mg/dL in EAs (Fig. S1a). ApoE level was significantly higher in females than males (*P* = 1.18E-24; Fig. S1b) and exposure to Ginkgo biloba showed no impact on plasma ApoE level (*P* = 0.769; Fig. S2).

To determine the association of plasma ApoE level with risk of incident dementia, we obtained hazard ratios (HRs) per 1-standard deviation (SD) lower ApoE level in 3031subjects using the Cox regression model adjusted for baseline age, sex, ethnicity, education, BMI, and the research site. For cognitive function, differences in cognitive scores per 1-SD decrease ApoE were obtained for subscale of the Alzheimer Disease Assessment Scale (ADAS-cog) and Modified Mini-Mental State Examination (3MSE) in all subjects from linear regression using the same covariates. While lower ApoE concentration in whole plasma was not associated with either incident dementia (HR = 1.00; 95% CI: 0.91–1.10) or AD (HR = 1.01: 95% CI: 0.92 to 1.12), it was associated with higher ADAS-cog scores, indicating worse cognitive function (*β* coefficient = 0.08; 95% CI: 0.01 to 0.18). A similar, but non-significant, association of lower ApoE concentration was observed with lower 3MSE scores, indicating worse cognitive function (*β* coefficient = −0.13 95% CI: −0.29 to 0.02).

Plasma ApoE is associated with multiple lipoprotein particles, which also contain other apolipoproteins, and about 50% of ApoE is present on high-density lipoprotein (HDL) [20]. Given its important role in lipid metabolism and AD dementia, it is possible that the association of ApoE level with dementia or cognitive function is modulated by its association with other apolipoproteins. To address this question, plasma lipoprotein-lipid along with HDL subfractions were determined in subset of the GEM sample comprising 1351 subjects [21]. While no association of baseline ApoE present in non-HDL or HDL particles was detected with incident dementia, lower ApoE level was significantly associated with higher ADAS-cog scores only in HDL (*β* = 0.20; 95% CI: 0.10 to 0.30) [21], as we also observed in this study in whole plasma in the total GEM sample of 3031 subjects. When this association in HDL was further examined in 1351 subjects based on the presence or absence of ApoC3 in HDL [21], this was confined to HDL lacking ApoC3 not only with ADAS-cog (*β* = 0.17; 95% CI: 0.07 to 0.27), but also with significantly lower 3MSE scores (*β* = −0.25; 95% CI: −0.42 to −0.07) as well as with incident and AD dementia (HR = 1.16; 95% CI: 1.03 to 1.32). These data showed that the presence or absence of ApoC3 in HDL modulates the association of plasma ApoE levels with dementia and cognitive function.

### Genome-wide association analysis

**Fig. 2:**
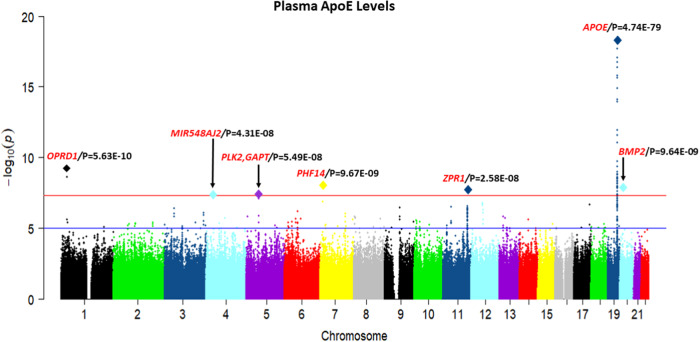
Manhattan plot of genome-wide association with plasma ApoE levels. The Red line depicts the genome-wide significance level (*P* < 5E-08) and the blue line represents suggestive associations (*P* < 1E-05).

Of 3031 subjects with plasma ApoE, DNA was available on 2737 participants (96.1% EAs) for genetic studies. Since the number of non-EAs was small, we included only EA participants in genetic analyses. A linear regression using the PLINK software was performed on 2580 EAs, including 2199 ND and 381 incident AD dementia cases (Table 1). A quantile-quantile plot did not demonstrate population stratification (λ = 1.005) (Fig. S3a). Six regions on chromosomes 1, 4, 7, 11, 19, and 20 showed genome-wide significant (GWS) signals (*P* < 5E-08) along with a subthreshold GWS signal (*P* = 5.49E-08) on chromosome 5 (Fig. 2).

**Table 1:** Baseline demographic information on 2580 European Americans non-demented and incident AD dementia participants.

	Cognitively Normal (*N* = 2199)	Incident AD Dementia (*N* = 381)	*P*
Male (%)	1227 (55.8%)	203 (53.3%)	0.391
Female (%)	972 (44.2%)	178 (46.7%)	
Age, years ± SD	78.27 ± 3.11	79.88 ± 3.65	4.57E-15
Mean Plasma ApoE mg/dL ± SD	4.11 ± 1.22	4.07 ± 1.26	0.77
Median Plasma ApoE mg/dL	3.9	3.8	
BMI, kg/m^2^ ± SD	27.21 ± 4.27	26.22 ± 3.82	6.44E-06
Education, years ± SD	14.23 ± 3.23	14.39 ± 2.84	0.37

### Associations in the *APOE* region

**Fig. 3:**
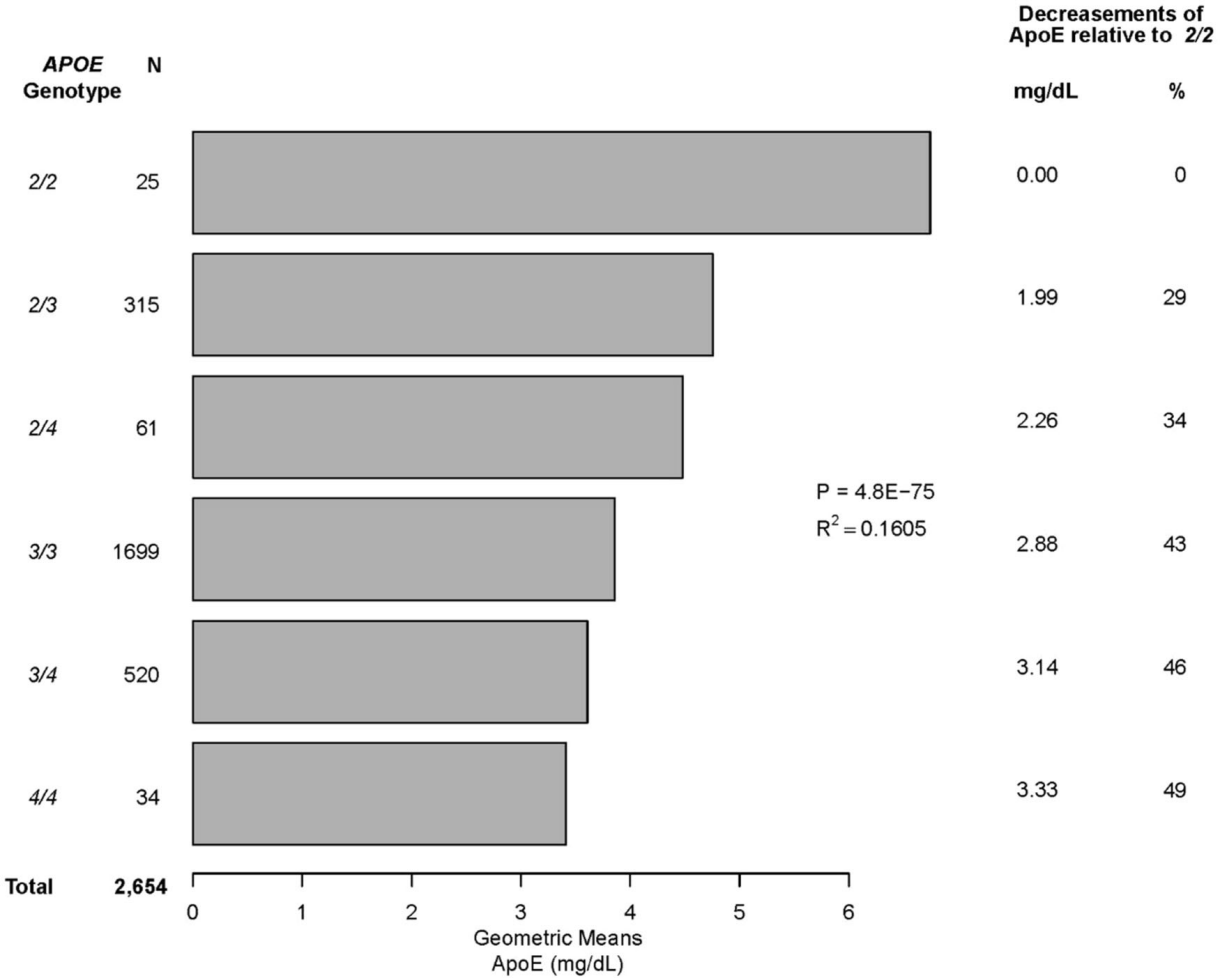
Plasma ApoE levels among six *APOE* genotypes. Values on the x-axis are the geometric mean of ApoE levels. R^2^ was adjusted for age and sex.

As expected, most of the significantly associated SNPs were from chromosome 19, where 57 SNPs surpassed the GWS threshold (Table S1; Fig. S3b). The most significant association was observed for APOE*2/rs7412, which was associated with elevating ApoE levels (*β* = 1.11; *P* = 4.73E-79). As expected, APOE*4/rs429358 was associated with lowering ApoE levels (*β* = −0.352; *P* = 8.73E-12). Since rs7412 and rs429358 correspond to the common *APOE* 2/3/4 polymorphism having six genotypes, we examined plasma ApoE levels among these genotypes (Fig. 3). The highest ApoE levels were observed in E2/2 homozygotes with a gradual decrease of 29%, 34%, 43%, 46%, and 49% in the 2/3, 2/4, 3/3, 3/4, and 4/4 genotypes, respectively.

There were several additional signals in the *APOE* region associated with increasing (42 SNPs) and decreasing (15 SNPs) plasma ApoE levels (Table S1). While APOE*2/rs7412 was the most significant SNP associated with higher ApoE levels, *APOC1P1*-*APOC4*/rs35136575 was the most significant SNP associated with lower ApoE levels (*β* = −0.3799; *P* = 6.34E-24), showing an even stronger effect than APOE*4. To determine which signals were independent, we conducted conditional analyses to find SNPs that were still significant after controlling for these two SNPs followed by an examination of linkage disequilibrium (LD) plots to identify representative SNPs in each cluster. Ten SNPs with positive-*β* remained significant (*P* range = 1.51E-02 to 1.69E-07) after adjusting for the effect of APOE*2 (Table 2). All SNPs with negative-*β* remained significant after adjusting for the effect of *APOC1P1-APOC4*/rs35136575, of which eight were still GWS (Table 2). All 15 SNPs with negative-*β* had essentially no LD with rs7412 (R^2^ = 0 to 0.06) and five of them remained GWS after adjusting for rs7412.

**Table 2:** List of 26 SNPs in the *APOE* region that remained significant after adjusting for the effects of rs7412 (top SNP associated with β-positive) or rs35136575 (top SNP associated with *β*-negative) in conditional analyses.

CHR	BP	SNP	GENE	LOC	MAF	BETA	SE	*P*-value	*P* after Conditioned on rs7412 (APOE*2)	*P* after Conditioned on rs35136575
19	45302504	rs148933445	*CBLC*	Intronic	0.017	0.7064	0.128	3.68E-08	1.51E-02	2.21E-07
19	45387459	rs12972156	*NECTIN2*	Intronic	0.128	−0.2881	0.0507	1.43E-08	2.63E-05	3.95E-07
19	45388130	rs34342646	*NECTIN2*	Intronic	0.133	−0.2834	0.0498	1.40E-08	2.45E-05	3.39E-07
19	45392254	rs6857	*NECTIN2*	UTR3	0.149	−0.2845	0.0475	2.37E-09	5.58E-06	1.22E-07
19	45394336	rs71352238	*TOMM40*	Promoter	0.131	−0.2916	0.05	6.31E-09	1.02E-05	1.14E-07
19	45396144	rs11556505	*TOMM40*	Exonic (p.Phe131Leu)	0.129	−0.2965	0.0508	6.06E-09	9.57E-06	1.08E-07
19	45408628	rs769446	*APOE*	Promoter	0.092	0.4942	0.0571	8.33E-18	6.91E-03	5.59E-15
19	45408836	rs405509	*APOE*	Promoter	0.468	−0.2961	0.0332	8.43E-19	4.23E-05	7.14E-17
19	45410002	rs769449	*APOE*	Intronic	0.098	−0.3443	0.0569	1.64E-09	5.91E-07	2.26E-11
19	45411941	rs429358 (APOE*4)	*APOE*	Exonic (p.Cys112Arg)	0.125	−0.3469	0.0516	2.21E-11	**3.01E-08**	1.53E-12
19	45412079	rs7412 (APOE*2)	*APOE*	Exonic (p.Arg158Cys)	0.08	1.119	0.0574	3.57E-79	NA	8.06E-74
19	45413576	rs75627662	*APOE*	Downstream	0.179	0.3647	0.0431	4.14E-17	1.55E-06	6.15E-15
19	45414451	rs439401	*APOE,APOC1*	Intergenic	0.373	−0.2862	0.0344	1.47E-16	8.22E-06	1.61E-09
19	45415935	rs7256200	*APOE,APOC1*	Intergenic	0.103	−0.3522	0.0562	4.17E-10	3.52E-07	3.25E-12
19	45416478	rs584007	*APOE,APOC1*	Intergenic	0.37	−0.2857	0.0345	2.09E-16	7.78E-06	2.28E-09
19	45416741	rs438811	*APOC1*	Promoter	0.211	0.2817	0.041	8.32E-12	1.69E-07	9.44E-10
19	45418790	rs5117	*APOC1*	Intronic	0.206	0.2884	0.041	2.57E-12	2.58E-07	4.31E-10
19	45423944	rs144311893	*APOC1,APOC1P1*	Intergenic	0.016	1.395	0.126	8.66E-28	4.85E-04	2.31E-26
19	45425460	rs157595	*APOC1,APOC1P1*	Intergenic	0.393	−0.2203	0.0344	1.76E-10	**1.16E-08**	2.11E-05
19	45426792	rs141622900	*APOC1,APOC1P1*	Intergenic	0.057	1.221	0.0678	1.74E-68	2.01E-06	6.18E-65
19	45430280	rs5112	*APOC1P1*	ncRNA_exonic	0.462	−0.3367	0.0335	2.36E-23	**1.01E-20**	3.79E-18
19	45431453	rs114448690	*APOC1P1*	ncRNA_intronic	0.088	0.7209	0.0583	3.39E-34	1.53E-06	5.92E-29
19	45431636	rs113345881	*APOC1P1*	ncRNA_intronic	0.102	0.5913	0.0559	1.32E-25	4.68E-04	2.79E-23
19	45431658	rs8106813	*APOC1P1*	ncRNA_intronic	0.49	−0.3296	0.0335	1.81E-22	**6.23E-19**	1.29E-16
19	45436657	rs28795074	*APOC1P1,APOC4*	Intergenic	0.084	0.7302	0.0599	2.57E-33	7.93E-06	3.98E-28
19	45439163	rs35136575	*APOC1P1,APOC4*	Intergenic	0.265	−0.3799	0.0373	6.34E-24	**1.09E-18**	NA

^*^Bold indicates those SNPs that remained genome-wide significant (GWS) after adjusting for the top APOE*2/rs7412 SNP, irrespective of their associated *β* values *CHR*: Chromosome *MAF*: Minor Allele Frequency. *NA*: Not applicable

Based on LD among these 26 SNPs (Fig. S4), we identified 10 independent signals, including five with lowering effect: *APOC4*/rs35136575(intergenic), *APOE*/rs429358(p.Cys112Arg), *APOE*/rs4055 09(promoter), *APOC1*/rs157595(intergenic) and *APOC1P1*/rs5112 (ncRNA-exonic); and five with elevating effect: *APOE*/rs7412 (p.Arg158Cys), *APOE*/rs769446(promoter), *CBLC*/rs148933445(intronic), *APOC1*/rs144311893(intergenic), and *APOC1P*/rs114448690 (ncRNA-intronic). An additional partially independent signal associated with lowering effect on ApoE level was driven by an LD block of 5 SNPs (rs12972156, rs34342646, rs6857, rs71352238, rs115 56505) in *PVRL2* (*NECTIN2*) and *TOMM40*. Three of these are potentially functional: *PVRL2*/rs6857(3’UTR), *TOMM40*/rs71352238 (promoter), *TOMM40*/rs11556505 (p.Phe131Leu). While *PVRL2*/rs6857 is in LD (R^2^ = 0.69) with APOE*4/rs429358, the other two are only in a moderate LD (R^2^ = 0.46– 0.48) with the latter (Fig. S4) and thus may represent a partial independent additional signal.

### Novel Associations

**Fig. 4:**
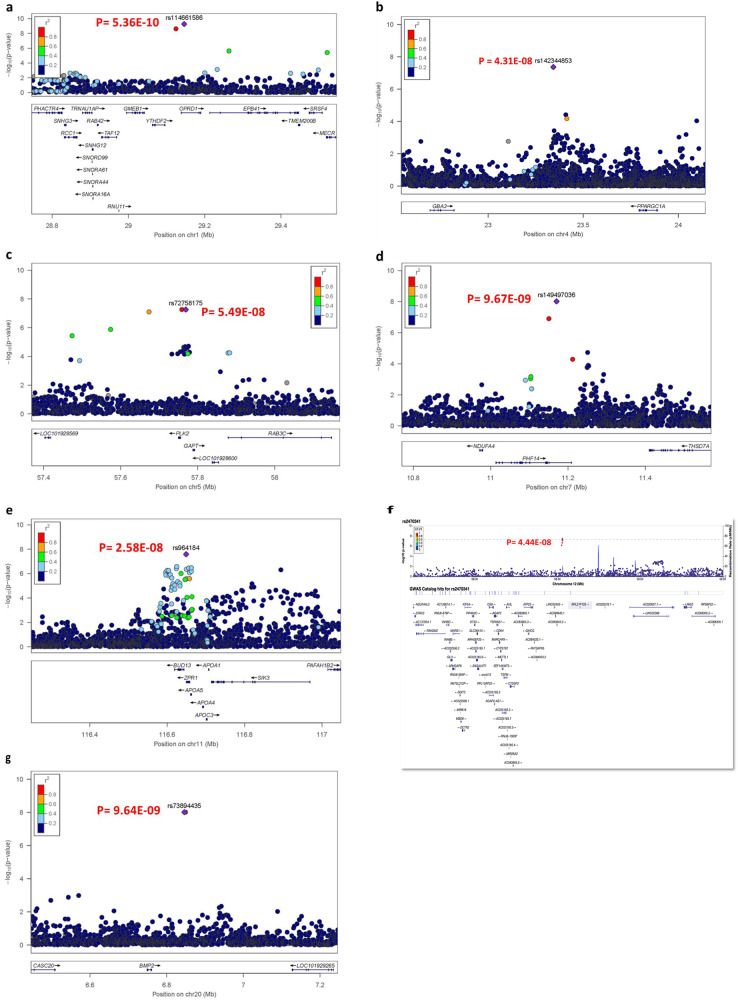
Regional plots of the association of plasma ApoE levels on chromosomes 1, 4, 5, 7, 11,12 and 20. **a** Regional plot in the *OPRD1* locus on chromosome 1; (**b**) Regional plot in the *GBA3,PPARGC1A* locus on chromosome 4; (**c**) Regional plot in the *PLK2* locus on chromosome 5; (**d**) Regional plot in the *PHF14* locus on chromosome 7; (**e**) Regional plot in the *ZPR1* (*ZNF259*)/*APOA5* locus on chromosome 11; (**f**) Regional plot in the *LINC02403* locus on chromosome 12 after adjusting for *APOC1P1,APOC4*/rs35136575; (**g**) Regional plot in the *BMP2* locus on chromosome 20.

In addition to known APOE*2/E*4 SNPs and the additional independent signals we discovered in the *APOE* region, we identified seven novel signals on chromosomes 1, 4, 5, 7, 11, 12 and 20; all were associated with elevated plasma ApoE levels (Table 3; Fig. 4**)**. While the two novel SNPs were genotyped on the chip (*ZPR1*/rs964184 and *BMP2*/rs73894435), the remaining were imputed. For this reason, we genotyped all carriers of the minor allele for the imputed SNPs using TaqMan assays and confirmed the imputed calls. The strongest novel signal, rs114661586, was present in intron 1 of *OPRD1* (*P* = 5.36E-10) followed by rs73894435 near *BMP2* (*P* = 9.64E-09), rs149497036 in intron 16 of *PHF14* (*P* = 9.67E-09), rs964184 in 3’UTR of *ZPR1/ZNF259* (*P* = 2.58E-08) and rs142344853 near *GBA3,PPARGC1A* (*P* = 4.31E-08). A subthreshold GWS signal observed on chromosome 5 near *PLK2, GAPT* (rs72758175; *P* = 5.49E-08) became GWS (*P* = 4.53E-09) after adjusting for APOE*2/rs7412. On the other hand, the chromosome 20 signal lost its GWS after adjusting for APOE*2/rs7412 (*P* = 2.21E-05), indicating a possible interaction between the two. However, we found no significant interaction between the two SNPs (*P* = 0.0661), indicating a biological rather than a statistical interaction that impacts plasma ApoE levels. After adjusting for *APOC1P1-APOC4*/rs35136575, an additional novel signal was observed on chromosome 12 near *AVIL-TSFM*/rs2470341 (*P* = 4.44E-08).

**Table 3:** Novel loci associated with plasma ApoE levels in addition to the *APOE* locus.

CHR	Position (GRCh37)	Gene	MAF	Lead variant	Consequence	A1	A2	BETA	*p*-value	*p*-value after adjusting for rs7412 (APOE*2)	*p*-value after adjusting for rs35136575
1p35.3	29146455	*OPRD1*	0.013	rs114661586	Intronic (Intron 1)	A	G	0.92	5.36E-10	3.51E-10	1.537E-09
4p15.2	23343005	*GBA3,PPARGC1A*	0.013	rs142344853	Intergenic	C	T	0.81	4.31E-08	4.456E-07	1.432E-07
5q11.2	57769068	*PLK2,GAPT*	0.024	rs72758175	Intergenic	G	C	0.62	5.49E-08	4.53E-09	2.697E-08
7p21.3	11170450	*PHF14*	0.017	rs149497036	Intronic (Intron 16)	G	A	0.72	9.67E-09	5.16E-08	1.696E-07
11q23.3	116648917	*ZPR1/ZNF259*	0.14	rs964184	3’ UTR (Exon 14)	G	C	0.27	2.58E-08	3.13E-09	1.29E-08
12q14.1	58530833	*AVIL-TSFM*	0.01	rs2470341	Intergenic	C	G	1.18	4.64E-07	4.94E-05	4.44E-08
19q13.32	45412079	*APOE*	0.08	rs7412	Arg158Cys	T	C	1.11	4.73E-79	_	8.059E-74
20p12.3	6846108	*BMP2*	0.013	rs73894435	Intergenic	A	T	1.37	9.64E-09	4.61E-05	9.738E-08

*CHR*: Chromosome, *MAF*: Minor Allele Frequency, A1: Effect minor Allele, A2: Major Allele

On chromosome 11, in addition to the top SNP *ZPR1*/rs964184, we also observed multiple suggestive associations (*P* range= 9.56E-07 to 2.62E-07), which remained significant after conditioned on the top SNP (Table S2). After considering the LD pattern between these SNPs and the top signal (Fig. S5), we identified three additional independent signals: *BUD13*/rs180326 (*P* = 2.62E-07), *SIK3*/rs4936359 (*P* = 4.85E-07); and *ZPR1*/rs35120633(p.A264V) (*P* = 3.48E-07).

### Estimation of plasma ApoE levels variance by *APOE* and non-*APOE* loci

The variance explained by a linear regression model regressing on age, sex and 11 independent *APOE* SNPs, described above, was 21.97% (*P* = 4.3E-31). The model with age, sex, and the 10 non-*APOE* SNPs (*OPRD1*/rs114661586, *GBA3-PPARGC1A*/rs142344853, *PLK2*/rs72758175, *ZPR1*/rs964184, *PHF14*/rs149497036, *BUD13*/rs180326, *SIK3*/rs4936359, *ZPR1*/rs35120633, *LINC02403*/rs2470341, *BMP2*/rs73894435) explained 9.26% (*P* = 4.6E-27) of the variance. Age and sex alone explained 4% (*P* = 8.7E-26) of the variance. The cumulative variance explained by both the *APOE* and non-*APOE* SNPs is 25.36% (*P* = 2.2E-32).

### Gene-based association analysis

**Fig. 5:**
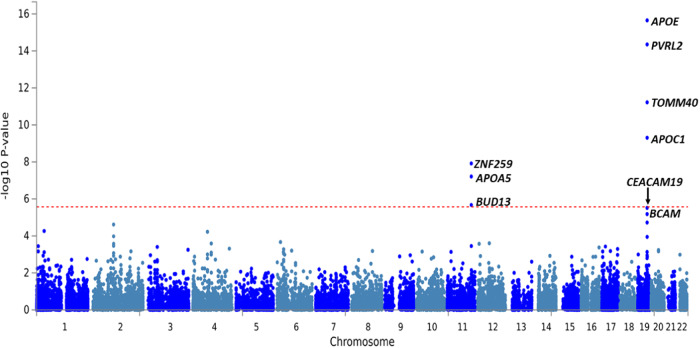
Manhattan Plot of gene-based test computed by MAGMA. The top genes are annotated. The Red line indicates the gene-wide significant threshold of *P* = 2.68E-06. *ZNF259* (*ZPR1*); *PVRL2* (*NECTIN2*). *APOE* (*P* = 3.16E-15), *PVRL2* (*P* = 4.44E-15), *TOMM40* (*P* = 5.98E-12) *APOC1* (*P* = 4.94E-10), *ZPR1*/*ZNF259*, (*P* = 1.21E-08), *APOA5* (*P* = 6.13E-08), *BUD13* (*P* = 2.12E-06), *CEACAM19* (*P* = 3.05E-06) and *BCAM* (*P* = 6.59E-06).

We conducted a gene-based association test using MAGMA (Multi-marker Analysis of GenoMic Annotation), which employs multiple linear regression on the full GWAS input data. The gene-wide significant threshold was set at *P* = 2.68E-06 (0.05/18,656 tested genes). A total of seven genes passed the gene-wide threshold, including *APOE*, *PVRL2*, *TOMM40*, *APOC1* on chromosome 19 and *ZPR1*/*ZNF259*, *APOA5*, *BUD13* on chromosome 11(Fig. 5). Two additional genes on chromosome 19 achieved subthreshold significance: *CEACAM19* and *BCAM*. These results provide further credence to the single-variant analyses on chromosomes 19 and 11.

### Functional bioinformatics analyses

To examine the biological significance of the identified variants and genes, we used the Functional Mapping and Annotation (FUMA) web-based platform (https://fuma.ctglab.nl/) to annotate, prioritize, visualize, and interpret GWAS results. FUMA has two core processes, SNP2GENE and GENE2FUNC [22]. SNP2GENE annotates SNPs for functional consequences on gene functions using ANNOVAR, deleteriousness score (CADD score), potential regulatory functions (RegulomeDB score) and effects on gene expression using expression quantitative trait loci (eQTL) and then mapped them to genes based on their physical position on the genomes, eQTL associations and 3D chromatin interactions. GENE2FUNC annotates the identified genes in biological context (gene expression, enrichment of differentially expressed genes in certain tissues, overrepresentation of gene sets, and general biological functions of input genes in term of their reported disease associations and drug targets).

A total of 116 pre-defined SNPs with GWS or suggestive associations (Table S3) with ApoE levels were used as input to SNP2GENE that mapped to 79 coding genes, including 49 in the novel regions and 17 in the *APOE* region (Table S4). Of the 79 genes, 77 had unique Entrez IDs which were further annotated to identify their gene expression and possible biological roles using GENE2FUNC that identified groups or pathways enriched for these 77 genes. *APOE* along with other genes in this region have relatively high expression in the brain (Fig. S6). Of the novel loci, no expression data were available for *PPARGC1A, GBA3*, and *BMP2* genes in GTEx. While the brain expression of *OPRD1, PLK2, PHF14* and *ZPR1/ZNF259* was modest, multiple candidate genes on chromosomes 11 and 12 showed high expression. As expected, based on the known roles of ApoE level and *APOE* genetic variation in lipid metabolism and AD, overrepresentation of gene sets, and general biological function analyses implicated lipid- and AD-associated pathways (Fig. S7, Table S5). Noteworthy, genes at novel loci were implicated with delirium on chromosome 11 (*P* = 3.15E-08) and hippocampal volume on chromosomes 11 and 12 (*P* = 3.50E-14; Table S5).

### Association of plasma ApoE level with AD-associated SNPs

Within the *APOE* region, Jansen et al. [23] identified 8 independent SNPs, in addition to APOE*4 and APOE*2, to be associated with AD risk. All these SNPs showed the expected association with plasma ApoE level in our study where AD risk allele was associated with lower ApoE level and AD protective allele with higher ApoE level (Table S6). Next, we examined the top 21 non-*APOE* AD loci [5] with ApoE level. Since the reported top SNP in one GWAS may not be the same in another GWAS, we examined multiple SNPs around the top reported AD-associated SNP in a given region. The top SNP in each AD region in our plasma ApoE GWAS was associated at nominal significance with ApoE level (Table S7) with the strongest association observed in the *ABCA7* region (*P* = 9.88E-05). We further checked if additional common and low-frequency variants within 17 AD-associated genes implicated by rare variants are associated with plasma ApoE level; all of them were nominal significant (Table S8). We acknowledge that some of these associations may be false-positive, but nevertheless they show a consistent pattern of association.

We also examined the ApoE level-associated novel SNPs in the *APOE* and non-*APOE* regions with AD risk and amyloid deposition in the brain (Table S9). All but two SNPs (rs35136575, rs114448690) in the *APOE* region were associated with AD risk in the IGAP discovery data [24]. Only 5 of the 12 SNPs in the *APOE* region were present in the amyloid-PET GWAS data [25] and all 5 were also associated with amyloid deposition. The non-*APOE* lead SNPs were not significant in the IGAP 2019 data. This is probably due to the low frequencies of all (MAF = 1–2%), but one variant. These complementary data suggest that at least a part of the AD genetic risk and amyloid deposition is mediated by genetically determined plasma ApoE variation, especially in the *APOE* region.

## Discussion

The main objective of this study was to expand the genetic basis of plasma ApoE variation by discovering new loci and additional signals in the *APOE* region. To our knowledge, this is the first large genome-wide analysis of plasma ApoE level. The secondary objective was to examine if baseline plasma ApoE level predicts cognitive function and incident dementia in a longitudinal and older cohort of GEM.

Earlier association studies of plasma ApoE level with AD have reported inconsistent findings [26–31], however, recent population-based epidemiological studies suggest that low plasma ApoE level may be a risk factor for incident dementia [14, 15]. In the current study, the baseline whole plasma ApoE level was not associated with incident dementia, but it showed a modest association with cognitive function. However, in subset of the GEM sample with lipoprotein subspecies data, the association of ApoE level in HDL with incident dementia and cognitive function was found to depend on the presence or absence of ApoC3 [21]. Baseline low plasma ApoE level present in HDL lacking ApoC3 was a risk factor for incident dementia and worse cognitive function, but not in HDL having ApoC3. A similar inverse relationship between plasma ApoE level in HDL lacking ApoC3 and risk of coronary heart disease (CHD) was reported in a large prospective population-based study [32], indicating that ApoE and ApoC3 on HDL interact to affect the risk of AD and CHD. These data suggest that ApoE present in HDL subspecies rather than in whole plasma is more relevant to estimating dementia risk and cognitive function. This may explain the discrepant results of the reported association studies of ApoE level with dementia. If this observation is confirmed in other studies that found association of low ApoE level with dementia without accounting for the role of HDL subspecies, it may provide new insight into the role of ApoE level in affecting the risk of AD and all-cause of dementia. While ApoE promotes metabolic steps in reverse cholesterol by rapidly clearing HDL from circulation when present on HDL in the absence of ApoC3, coexisting of ApoC3 with ApoE on HDL attenuates this beneficial effect on CHD [32]. A similar mechanism may occur in the brain where lipoproteins are HDL-like [33] and low concentration of ApoE on HDL lacking ApoC3 may affect the neuronal cholesterol uptake and clearance of Aβ and tau mediated by ApoE-binding receptors and eventually affecting cognitive function and AD risk.

The genome-wide analysis identified seven new loci in addition to the known *APOE* locus. We confirmed the established and opposite associations of APOE*2/rs7412 and APOE*4/rs429358 with plasma ApoE level. Surprisingly, APOE*4 was not the top SNP in lowering ApoE level; rather, we identified a novel signal, *APOC1P1*-*APOC4/*rs35136575, associated with lower level (*P* = 6.34E-24). Among the 57 GWS SNPs detected in the *APOE* region, ten independent and one partial independent signal was identified, including five with elevating effect and six with lowering effect. Four of these signals are present in the *APOE* gene, indicating its primary role in affecting plasma ApoE level. This is further confirmed in the gene-based analysis where *APOE* was gene-wide significant. Four signals are intergenic between *APOC1* and *APOC4*; *APOC1* was also gene-wide significant. One partial signal may be mediated by coding or a promoter SNP in the *TOMM40* gene, which was also gene-wide significant. The single-variant and gene-based analyses suggest that *APOE*, *APOC1*, *TOMM40* and *PVRL2* (*NECTIN2*) are the likely genes affecting plasma ApoE level in this region. Altogether, the 11 independent SNPs in the *APOE* region explained about 22% of the variance of plasma ApoE level. By comparison, the seven non-*APOE* new signals explained 9% of the variance.

In the *APOE* region, association of ApoE2 with high ApoE level is due to its decreased binding with LDL receptor, resulting in higher plasm level. While the association of ApoE4 with lower ApoE level is thought to be i) its preference binding with very-low density lipoprotein as compared to the preference binding of E2 and E3 with HDL, causing its accelerate hepatic clearance or ii) more likely, it is the result of misfolding and accumulation of ApoE protein in endoplasmic reticulum due to an ionic interaction between Arg-61 in the N-terminal domain and Glu-255 in the C-terminal domain in the E4 isoform; this domain interaction is not present in the other two ApoE isoforms [3, 4]. The independent associations of two *APOE* promoter SNPs (rs769446, rs405509) with ApoE level are most likely due to their close proximity to LXR/RXR response element, as *APOE* expression is regulated by LXR/RXR. LXR is a member of a group of nuclear receptors, peroxisome proliferator-activated receptors (PPARs; other members are PPARγ and PPARδ), each of which upon activation heterodimerizes with retinoid X receptor (RXR) to form a functional transcription factor along with coactivators that act to regulate expression of many genes, including those involved in AD pathogenesis [34]. Based on RegulomeDB functional annotation where lower scores (range = 1a to 7) strongly indicate regulatory functions, two additional independent signals can likely affect ApoE level. *APOC4*/rs35136575 with a RegulomeDB score of 1d, can affect transcription binding. Another independent signal represented by three LD-linked SNPs (rs157595, rs439401 and rs584007) have RegulomeDB scores of 1f, 1c, and 1f, respectively, indicating that these SNPs have regulatory functions as they can affect transcription binding and expression of a gene target.

Three of the seven novel SNPs in our study also implicate genes in the activation of PPARs/RXR. The chromosome 4 intergenic signal, rs79399931, is located between *GBA3* and *PPARGC1A*. *GBA3* is involved in glucose metabolism pathways and its genetic variation has been reported to be associated with fasting glucose and insulin levels [35]. *PPARGC1A* codes for PPARγ coactivator-1-alpha (PGC-1α), which is a transcriptional coactivator. It is possible that *PPARGC1A* genetic variation, which is also associated with Parkinson’s disease, CHD, and type 2 diabetes [36–38] can affect its binding with target transcription factors and thus expression of many genes, including *APOE*.

The chromosome 11 signal, rs964184, is located in 3’UTR of *ZPR1*/*ZNF259* and has been associated with triglycerides [39, 40], type 2 diabetes [39] and abdominal aortic aneurysm (AAA) [41]. Functional annotation of this variant showed a RegulomeDB score of 1f, indicating a high degree of evidence for being a regulatory variant that can affect transcription binding and gene expression. Since the submission of our manuscript, *ZPR1*/rs964184 has been implicated in the LXR/RXR activation pathway that affected the expression and plasma level of ApoE, along with ApoA1, ApoA5 and ApoC3 [41]. This provides confirmation to our independent observation and affirms the association of *ZPR1*/rs964184 with ApoE level being genuine. *ZPR1*/*ZNF259* promoter region has the binding sequence for PPARs, which may activate genes involved in glucose and cholesterol metabolism, including *APOE*, via hepatocyte nuclear factor 4 alpha activation [39]. In addition to *ZPR1*/rs964184, we identified three additional independent suggestive signals in this region affecting plasma ApoE level, and one of them is characterized by two coding SNPs: *APOA5*/rs3135506 (p.Ser19Trp), *ZPR1*/rs35120633 (p.Ala264Val) and a regulatory intronic SNP, *ZPR1*/rs12285095 (RegulomeDB score = 2b).

The chromosome 20 signal, rs73894435, is located near *BMP2* that codes for BMP2 (bone morphogenetic protein-2) which belongs to the family of bone morphogenetic proteins that act as regulators of growth and differentiation of several cell types. BMP2 was originally shown to upregulate *APOE* in a murine-mesenchymal progenitor cell line, suggesting a possible role of *APOE* in organogenesis and tissue differentiation [42]. The mechanism of BMP2-mediated upregulation of ApoE is operated through a novel PPARγ/ApoE axis down stream of BMP2 and its receptor, BMP-R2. The BMP2/BMP-R2 signaling leads to activation of PPARγ and the resulting production and secretion of ApoE [43]. Since *BMP2*/rs73894435 lacks evidence of being a regulatory variant (RegulomeDB score = 5), we hypothesize that this variant could be in LD with a coding variant in BMP2 that enhances its binding with BMP-R2, resulting enhanced BMP2/BMP-R2 signaling and the ensuing high production of ApoE associated with this variant.

Although we did not find any reported mechanisms for the association of other four novel variants with ApoE level, genes associated with these variants have been implicated with AD and autoimmunity. Chromosome 1 signal, rs114661586, is present in intron 1 of *OPRD1* that encodes delta-opioid receptors, which are implicated in cognitive functions [44] and elevated *OPRD1* promoter methylation is suggested to be a risk factor for AD [45]. Chromosome 5 signal, rs72758175, is located near *PLK2* (polo-like kinase 2) that binds and phosphorylates Thr-668 and Ser-675 residues of amyloid precursor protein, which instigate Aβ production in hippocampal neurons, suggesting that PLK2 may be an important target molecule for AD treatment [46]. Chromosome 7 signal, rs149497036, is in intron 16 of *PHF14*, whose function is not clear. However, the nearby gene, *NDUFA4*, that codes for a mitochondrial protein has been identified as one of the few significantly regulated proteins in Aβ plaques in anti-Aβ antibody aducanumab treated transgenic mice, suggesting that upregulation of NDUFA4 in plaques may inhibit Aβ toxicity and increase phagocytosis and cell viability [47]. Additionally, ApoE level was also increased by aducanumab within plaques core, suggesting a possible common mechanism by which NDUFA4 could affect ApoE level other than aducanumab. Chromosome 12 signal, rs2470341, is intergenic and located near the *AVIL-TSFM-CYP27B1-TSPAN31* locus, which is gene-dense and previously been implicated with multiple distinct autoimmune disorders [48]. Our variant is distinct since it was not in LD with any of the SNPs implicated in autoimmune disease. We hypothesize that the ApoE level-associated variants in these four novel loci may be in LD with SNPs that are located in regions having regulatory potential that directly or indirectly affect *APOE* expression. Future fine-mapping and functional studies would be helpful in delineating the ApoE level-associated mechanisms of these associations.

Important biological questions are whether and how genetically determined ApoE level can affect the risk of AD or dementia and whether the observed variation in plasma ApoE level is also reflected in the CNS. We show here that in addition to the established and opposite associations of APOE*4 and APOE*2 with both AD risk and ApoE level, multiple additional independent AD-associated SNPs reported in the *APOE* region [23] as well as reported SNPs in some known non-*APOE* AD loci [5], are also associated with plasma ApoE level. Similarly, multiple ApoE level-associated independent SNPs observed in the *APOE* region in this study were also associated with AD risk. These data suggest that variation in ApoE level is associated with AD risk, with a significant contribution from multiple signals in the *APOE* region. The murine data in targeted replacement *APOE* mice and *APOE* knock-in mice support this observation, wherein reduced brain ApoE level and reduced basal dendritic spine density in the entorhinal cortex were found in ApoE4 mice compared with ApoE3 mice [49]. Although the origins of ApoE in the CNS (mainly from astrocytes) and circulation (hepatocytes) are distinct [50], and there is a very low correlation between human plasma and cerebrospinal fluid (CSF) ApoE level, CSF level is under the similar genetic control of the *APOE 2/3/4* polymorphism as in plasma [16]. Similarly, ApoE level in the hippocampal interstitial fluid of mice has been shown to be dependent upon the *APOE 2/3/4* polymorphism [51]. ApoE deficiency in mice has also shown to be associated with BBB dysfunction where both the blood- and tissue-derived ApoE were found to be equally important for BBB function and thus relevant to age-related neurodegenerative diseases, like AD [52]. The above data provide credence to a possible molecular mechanism where genetically determined brain ApoE level could affect AD pathological changes. However, it is not clear how the brain ApoE, which is derived from different cells with a distinct function, affect the AD risk. For example, while the normal brain secretes most of the ApoE in HDL-like lipidated lipid particles from astrocytes, in neurodegeneration, microglia secrete ApoE in poorly lipidated lipid particles and the presence of ApoE in Aβ plaques is mainly derived from microglia. Similarly, while astrocyte-derived ApoE4 has a neuroprotective effect, neuronal-derived ApoE4 does not [50, 53].

The strength of our study is that we have used the largest sample size to date with 90% power to detect 0.0174 effect size index at GWS level, which has enabled the identification of multiple novel signals in the known *APOE* region as well as novel loci in the genome. These findings improve our understanding of the genetic control of plasma ApoE level and provide the potential for additional paths to amelioration of AD pathology. Some limitations of our study include the use of an older cohort and lack of replication sample. GWAS findings in this older cohort may not be generalizable, and some ApoE level-associated variants may have been missed.

## Conclusion

This is the largest GWAS on plasma ApoE level that, in addition to confirming the previously reported association of APOE*2 and APOE*4, has identified additional independent signals in the *APOE* region as well as seven new loci on other chromosomes affecting plasma ApoE level. We also found that the ApoE level-associated independent variants, especially in the *APOE* region, are associated with AD risk and amyloid deposition, indicating that genetically determined ApoE variation may be a risk factor for developing AD. Further longitudinal studies in independent samples may help to delineate the role of ApoE level in influencing AD risk.

## Online methods

### Participants

Study participants were from the GEM study, which was designed to test the effect of *Ginkgo biloba* on preventing/delaying the development of incident dementia. The study was approved by the University of Pittsburgh Internal Review Board and informed consent was obtained from all subjects. Participants were recruited from four clinical sites in the United States during 2000 to 2002. Individuals who already showed signs of dementia or those who had other neurological conditions were excluded from the study. A full description of recruitment, screening procedures, and outcomes in the GEM study has been reported elsewhere [17–19]. A flow chart of study participants included in different analyses is shown in Fig. 1. A total of 3069 community-residing volunteers aged 72 to 96 years (95.5% EAs) with normal cognition (*n* = 2587) or mild cognitive impairment (MCI; *n* = 482) at baseline were screened every 6 months for incident dementia until 2008. Five hundred twenty-three individuals developed incident dementia, of which 92% were classified as AD. Of the 3034 baseline plasma samples available, plasma ApoE levels were determined on 3031 participants (95.4% EAs, 4.6% other ethnicity); of which DNA was available on 2737 participants (96.1% EAs, 3.9% other ethnicity) for genetic studies. Since the number of other ethnicities was small, we included only EAs participants in subsequent analyses.

### Plasma ApoE levels

Baseline plasma ApoE levels were quantitatively measured using the Kamiya Biomedical Company’s ApoE assay, KAI-007 (Seattle, USA) following the manufacturer’s instructions. The assay uses polyclonal antibodies that react with all three ApoE isoforms (Apo E2, E3, and E4). The samples were run on an Olympus AU400 automated chemistry analyzer (Olympus Company Ltd., Tokyo, Japan) with two control samples from Kamiya Biomedical Company (K112C-4M). This immunoturbidimetric assay mixed the sample with an anti-human ApoE antiserum that causes agglutination. The resulting turbidity was then measured at 340 nm and 700 nm and the total amount of ApoE was determined quantitatively using the KAI-25C calibrator (KAIYU, Japan). All samples were run in duplicates to assess the quality of the assay. All duplicate samples showed comparable values.

### Genotyping and quality control (QC)

All 2737 participants having DNA were genotyped using Illumina Infinium Multi-Ethnic Global-8 v1.0 chip containing 1,748,250 single-nucleotide polymorphisms (SNPs) at the University of Pittsburgh Genomics Research Core. Genotypes for two *APOE* SNPs [rs429358 (APOE*4) and rs7412 (APOE*2)] were determined either as previously [54] described or using TaqMan® SNP genotyping assays (Applied Biosystems, ThermoFisher Scientific, USA). Participants with >2% genotype failure rate and cryptic relatedness (none removed) were used as exclusion criteria. Exclusion criteria for SNPs included minor allele frequency (MAF) <1%, high genotype failure rate, and deviation from Hardy–Weinberg expectations (*P* ≤ 1E–06). The top imputed SNPs with genome-wide significant P-values were genotyped using TaqMan assays (Applied Biosystems, Thermo Fisher Scientific).

### Imputation and population stratification

Genotype posterior probabilities were imputed using the Haplotype Reference Consortium (HRC) panel on the Michigan imputation server (https://imputationserver.sph.umich.edu/), which resulted in 14,072,053 QC passed SNPs for downstream analyses.

Population stratification was analyzed using a multi-dimensional scaling-based method as implemented in PLINK using only QC-passed (*n* = 5,448,855 SNPs) common variants (MAF > 0.05) [55]. For the estimation of correlation (R^2^), a sliding window method of 2000 bp shift after every 200 variants was applied. This conservative sliding window approach also prevented the incorporation of highly related genetic variants for the estimation of ethnicity structure. Variants with maximum likelihood phasing (R^2^ > 0.5) were detected and excluded for principal component analysis (PCA). The first four components were conservatively determined to be relevant for the determination of population origin based on the visual examination of the principal component (PC) of ancestry plots and were used as covariates in subsequent statistical association analyses.

### Statistical analyses

#### Plasma ApoE Level and *APOE* genotype analysis

To determine the association of plasma ApoE level with risk of incident dementia, we obtained hazard ratios (HRs) per 1-standard deviation (SD) lower ApoE level in 3031 subjects using the Cox regression model [21] adjusted for baseline age, sex, ethnicity, education, BMI, and the research site. For cognitive function, differences in cognitive scores per 1-SD decrease ApoE were obtained for subscale of the Alzheimer Disease Assessment Scale (ADAS-cog) and Modified Mini-Mental State Examination (3MSE) in all subjects from linear regression using the same covariates. To examine the effect of *APOE* genotype with plasma ApoE level, EA participants with the available genotype and plasma ApoE data were coded according to plasma ApoE level and six *APOE* genotypes (2/2, 2/3, 2/4, 3/3, 3/4, and 4/4*)*. Linear regression was used for the estimation of plasma ApoE levels based on the *APOE* genotypes while adjusting for sex and baseline age.

#### GWAS analysis and Power

SNP analysis was performed using a linear regression framework implemented in PLINK, including age, sex, education, and the first four PCs of ancestry as covariates. The genome-wide significance (GWS) threshold was set at *P* < 5E-08, while the suggestive significance threshold was set at *P* ﻿≤ 1E-06. Power was calculated using the G*Power software [56]. Our sample size of about 2600 subjects included in GWAS analysis has sufficient power to detect small variation; it has 90% power to detect 0.0174 effect size index (f^2^) at GWS level (*α* = 5E-08) that corresponds to 1.71% variance (R^2^) explained by a single SNP. The GWS detectable variance of 2.27% at 99% Power is still excellent.

### Functional annotations

SNPs were functionally annotated using FUMA of genome-wide association studies (FUMA-GWAS; https://fuma.ctglab.nl/) with known functional annotations, including RegulomeDB (RDB) scores, combined annotation dependent depletion (CADD) scores, chromatin states, and ANNOVAR. RDB scores range from 1a to 7, with lower scores strongly indicating regulatory functions. CADD scores predict the deleterious nature of a SNP and a score above 12.37 is potentially pathogenic. The chromatin state represents the accessibility of genomic regions having 15 categorical states, with a lower state indicating higher accessibility; chromatin states 1–7 reflect open chromatin states. ANNOVAR annotates SNPs for their locations in genes (intronic, exonic, or intergenic). SNPs with GWS associations and *P* ≤ 1E-06 were used as pre-defined lead SNPs in the SNP-GENE resource of the FUMA-GWAS web server. This analysis mapped genes using functional consequences of SNPs on genes. SNPs were annotated to a specific gene if they were located within or 1 kb upstream of the transcriptional binding site or 1 kb downstream of the transcriptional ending site of the gene. Genes successfully mapped to significant SNPs were further annotated in biological context using the GENE-FUNC function of FUMA-GWAS. Tissue expression of all top genes and subthreshold genes resulted from a gene-based test (MAGMA on FUMA-GWAS) in the brain and blood was fetched from an online resource (https://www.proteinatlas.org). The sentinel SNPs associated with plasma ApoE levels were further investigated in the QTL database [57] (http://www.mulinlab.org/qtlbase) to get eQTL information.

### Supplementary information

Genome-wide analysis identifies novel loci influencing plasma apolipoprotein E level
