# Supplementary material for: Genome-wide analysis identifies novel loci influencing plasma apolipoprotein E concentration and Alzheimer’s disease risk
Source: Mol Psychiatry. 2023 Sep 5;28(10):4451–62. doi: 10.1038/s41380-023-02170-4 (PMC10827653; doi:10.1038/s41380-023-02170-4)
Supplement: Supplementary file 1 — Genome-wide analysis identifies novel loci influencing plasma apolipoprotein E level [file 41380_2023_2170_MOESM1_ESM.docx]

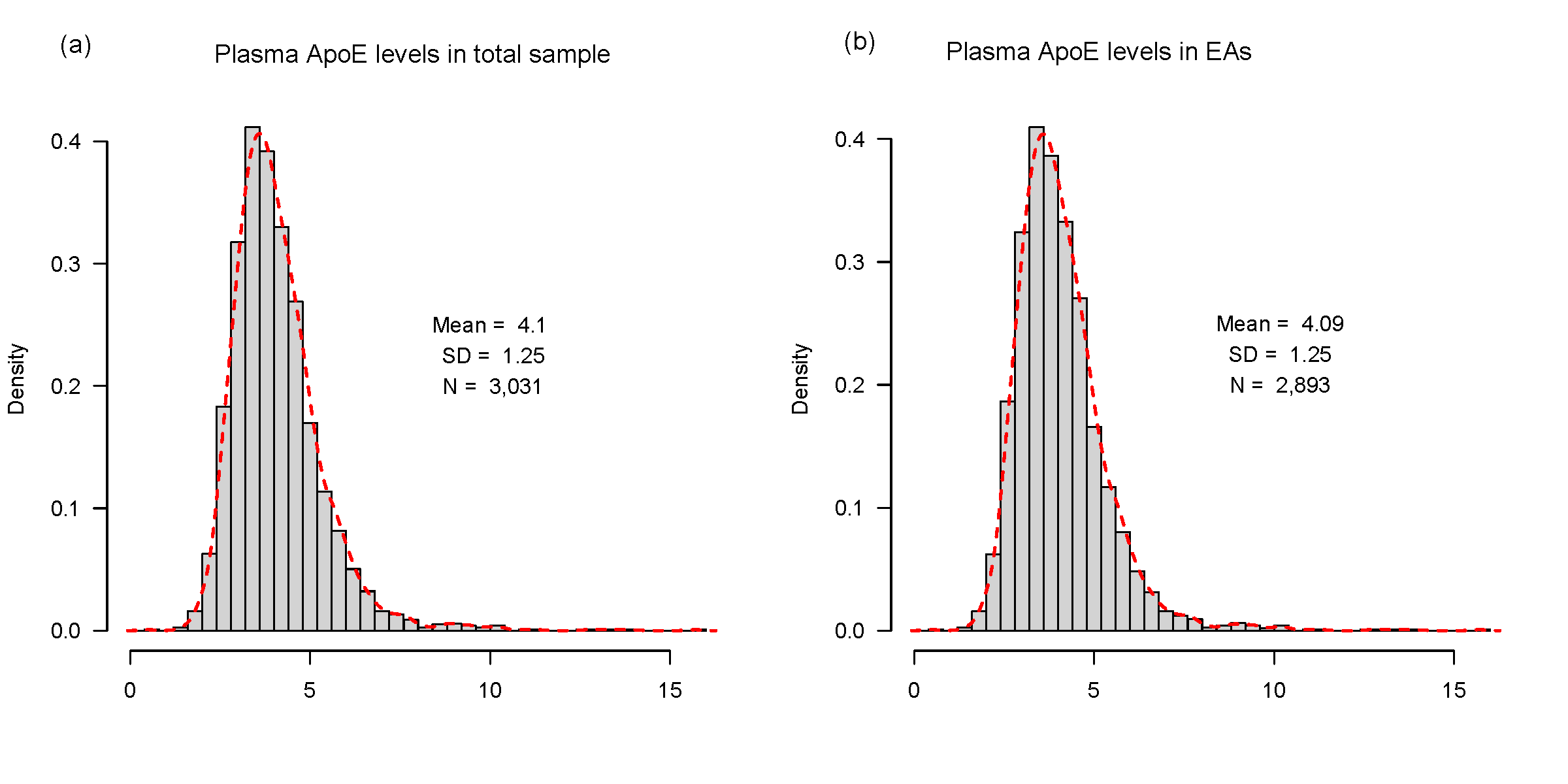


**Supplementary Figure S1a:** Distribution of plasma ApoE levels in the total sample of 3,031 and 2,893 European Americans (EAs)

**Supplementary Figure S1b:** Distribution of plasma ApoE levels in men and women in the total sample of 3,031 and 2,893 European Americans (EAs).


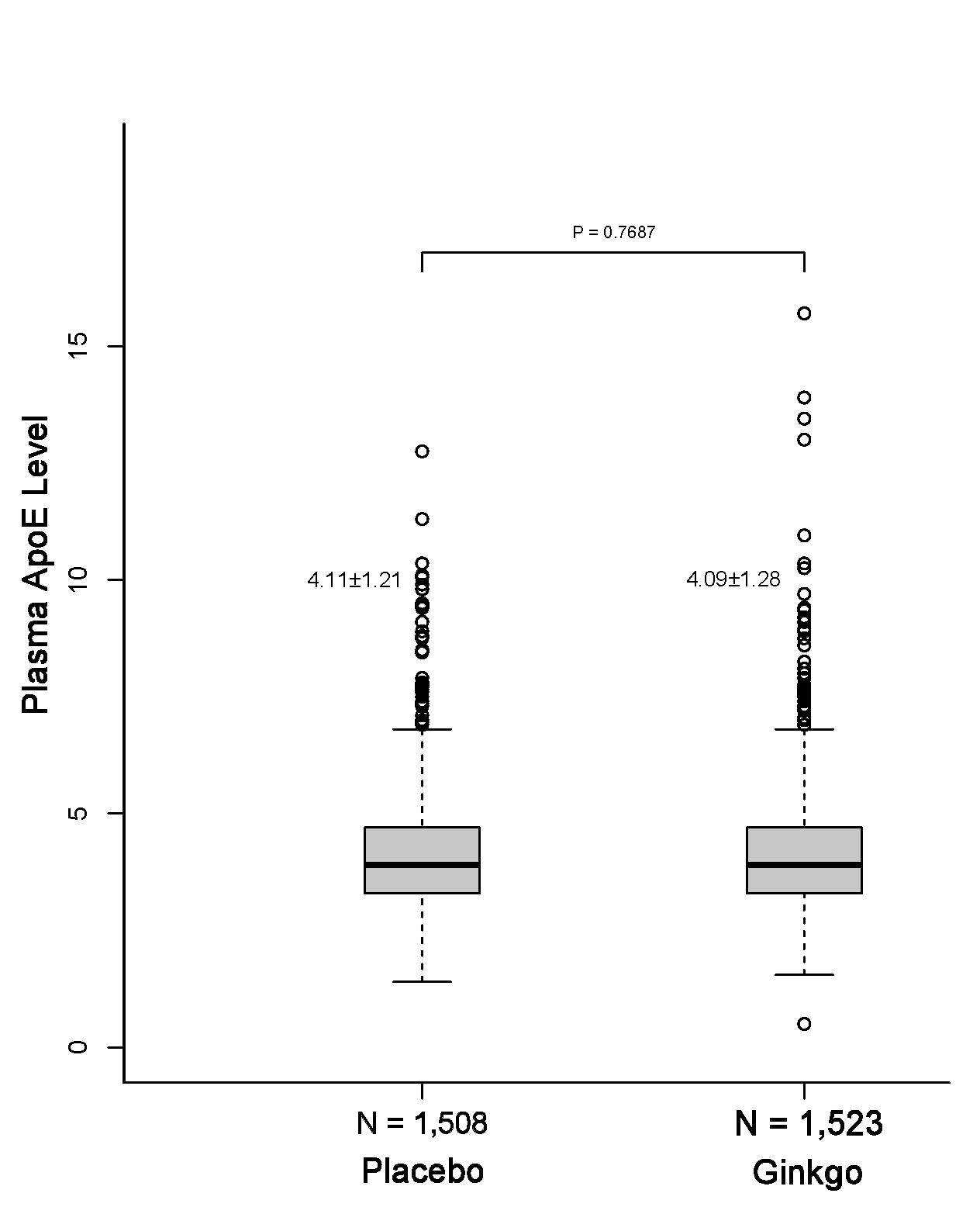


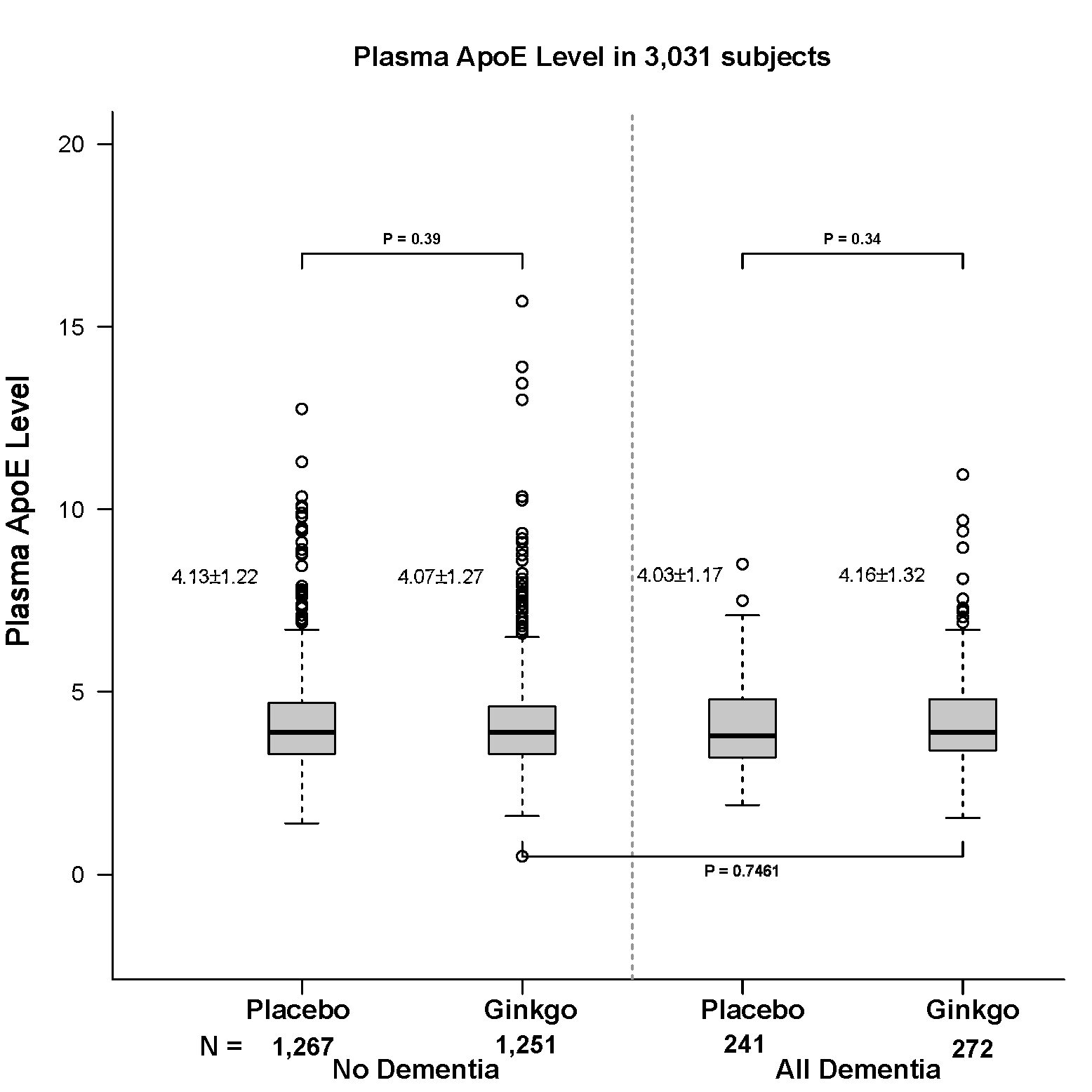


**Supplementary Figure S2: Top:** Distribution of plasma ApoE level between the Placebo and Ginkgo biloba groups in 3,031 subjects. **Bottom:** Distribution of plasma ApoE level between the Placebo and Ginkgo biloba groups stratified by Dementia and No Dementia groups

**Supplementary Figure S3: a)** QQ-plot showing the comparison of expected versus observed p-values including all QC-passed SNPs. The red line shows the distribution under the null hypothesis; **b)** Regional plot of the association of plasma ApoE levels in the *APOE* region.


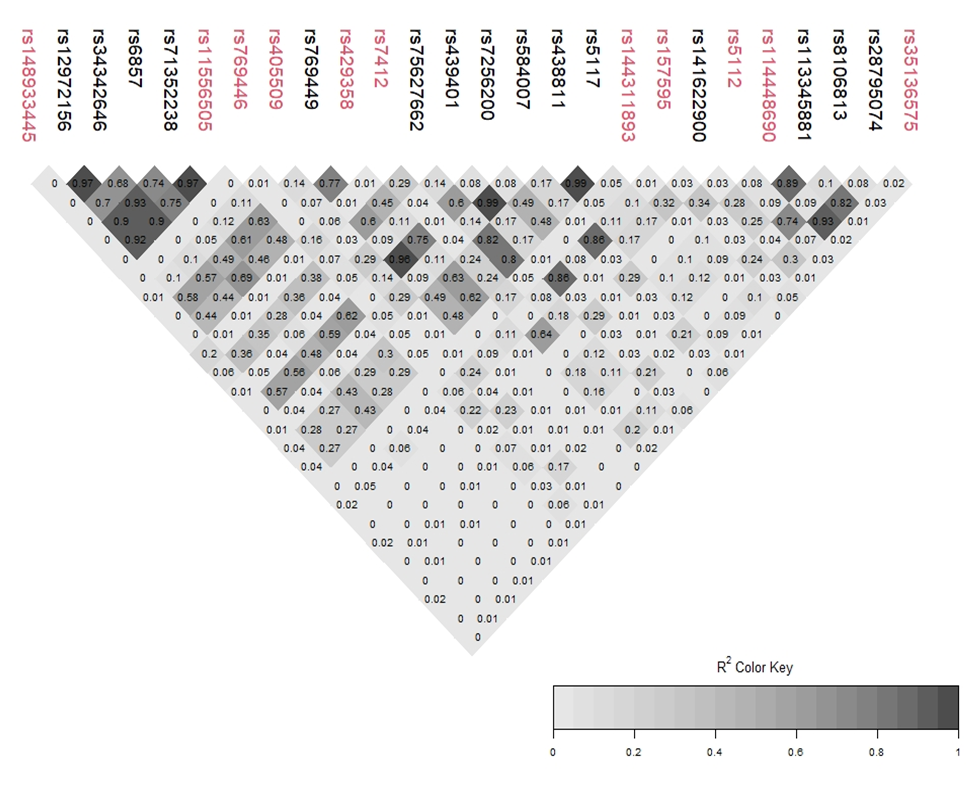


**Supplementary Figure S4:** LD between SNPs in the *APOE* region on chromosome 19 that remained significant after adjusting for the effects of rs7412 (top SNP associated with β-positive) and rs35136575 (top SNP associated with β-negative) in conditional analyses. Red color indicates the independent signals in this region.


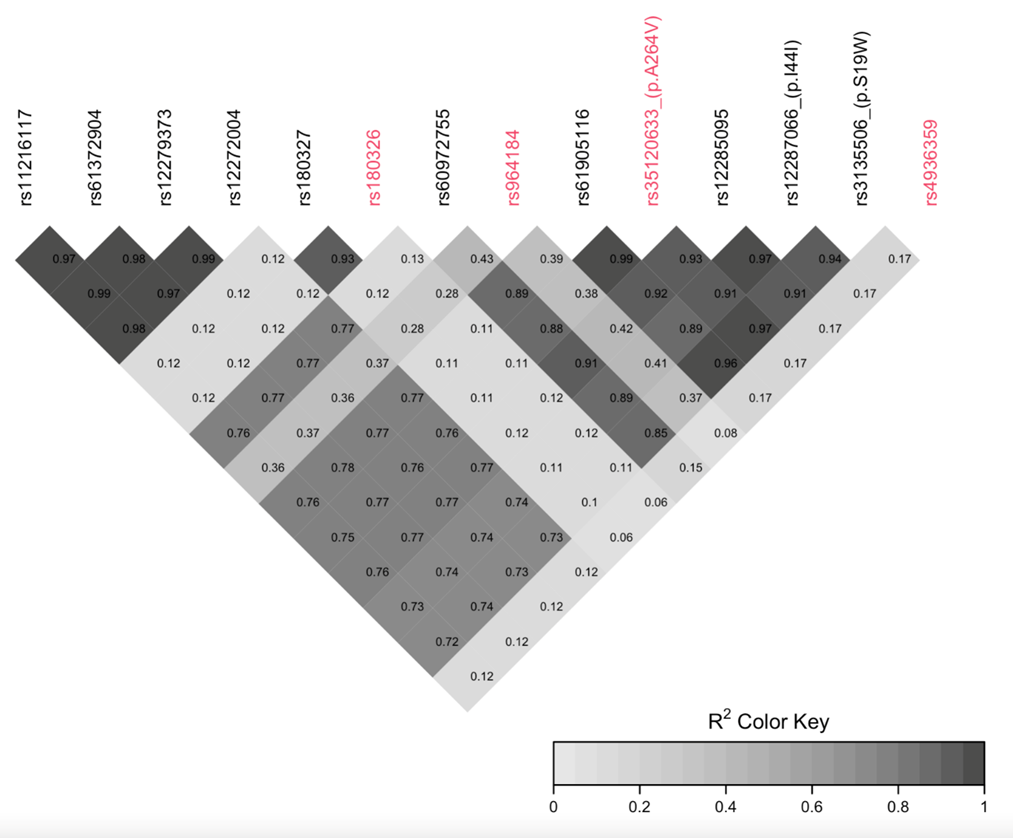


**Supplementary Figure S5:** LD between SNPs in the *ZPR1*/*ZNF259* region on chromosome 11, including the top genome-wide significant SNP (rs964184) and suggestive SNPs (P range = 9.56E-07 to 2.62E-07). Red color indicates the independent signals in this region.

f

e


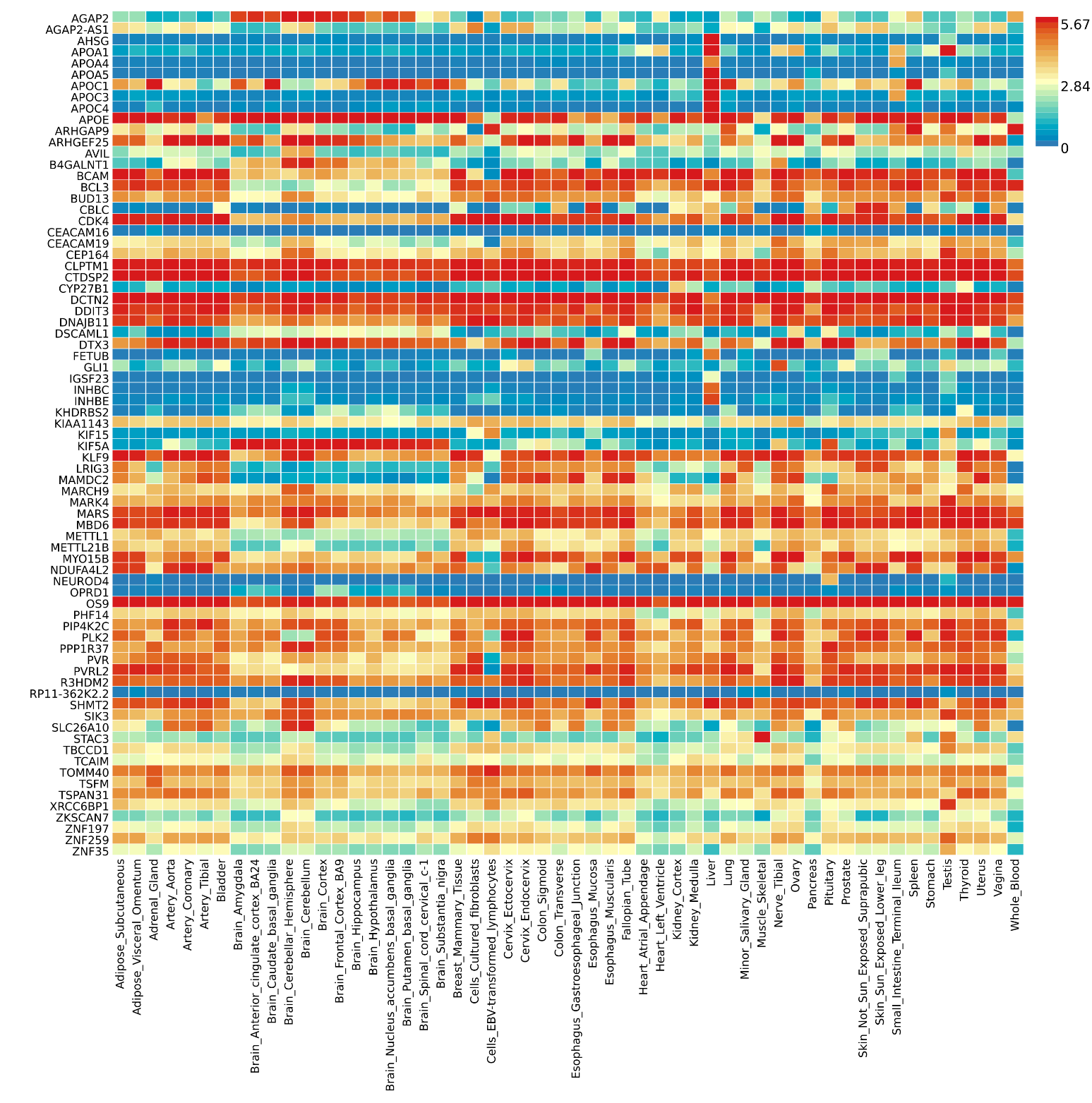


**Supplementary Figure S6:** Full tissue-specific gene-expression heat map from GTEx V8 54 tissue types of mapped genes.


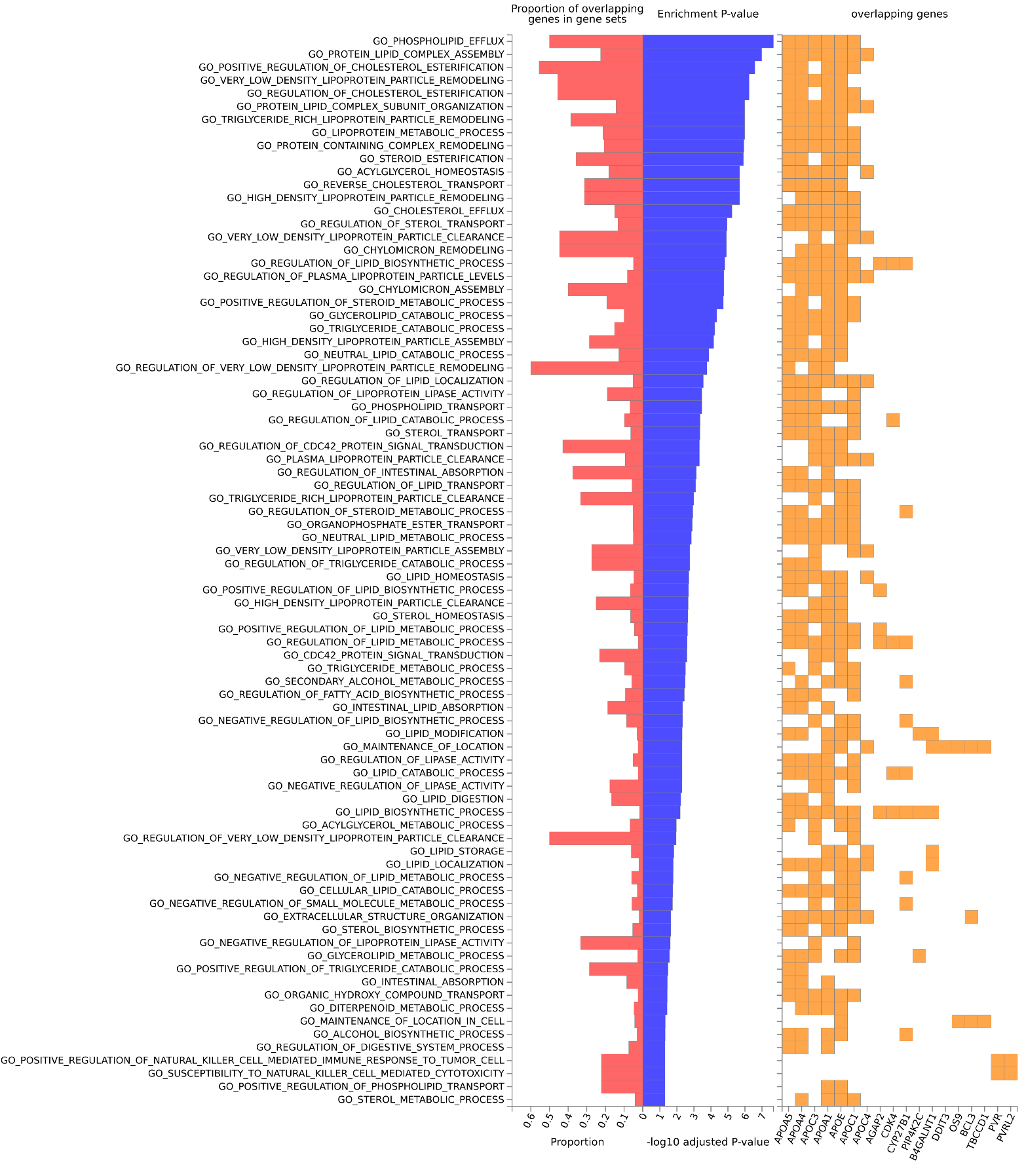


**Supplementary Figure S7:** GO biological process (MsigDB5) showing the enrichment of genes in different biological pathways.

**Supplementary Table S1:** List of 57 SNPs that surpassed the genome-wide significant threshold at chromosome 19. SNPs in red are those with negative beta-values; the rest are with positive beta-values.

| **CHR** | **Position (GRCh37)** | **SNP** | **A1** | **A2** | **Consequence** | **GENE** | **MAF** | **BETA** | **P-value** | **Conditioned on rs7412 (APOE*2)** | **Conditioned on rs35136575** |
| --- | --- | --- | --- | --- | --- | --- | --- | --- | --- | --- | --- |
| 19 | 45232161 | rs62117160 | A | G | Intergenic | *CEACAM16,BCL3* | 0.0351 | 0.69 | 1.03E-14 | 8.02E-01 | 8.14E-14 |
| 19 | 45242173 | rs1531517 | A | G | Intergenic | *CEACAM16,BCL3* | 0.0678 | 0.39 | 1.47E-08 | 5.21E-01 | 2.02E-07 |
| 19 | 45242740 | rs2927439 | G | A | Intergenic | *CEACAM16,BCL3* | 0.3412 | 0.20 | 1.28E-08 | 7.14E-02 | 2.80E-06 |
| 19 | 45242967 | rs62117204 | T | C | Intergenic | *CEACAM16,BCL3* | 0.0647 | 0.39 | 2.31E-08 | 4.30E-01 | 2.93E-07 |
| 19 | 45247627 | rs4803750 | G | A | Intergenic | *CEACAM16,BCL3* | 0.0649 | 0.41 | 3.29E-09 | 4.31E-01 | 8.18E-08 |
| 19 | 45249509 | rs115785198 | T | C | Intergenic | *CEACAM16,BCL3* | 0.0632 | 0.38 | 4.12E-08 | 2.85E-01 | 7.72E-07 |
| 19 | 45255266 | rs62117205 | C | T | Intronic | *BCL3* | 0.0639 | 0.40 | 5.86E-09 | 3.92E-01 | 1.53E-07 |
| 19 | 45295886 | rs113330691 | A | G | Intronic | *CBLC* | 0.0373 | 0.55 | 1.32E-09 | 2.76E-01 | 4.57E-10 |
| 19 | 45296364 | rs112450640 | A | G | Intronic | *CBLC* | 0.0186 | 1.06 | 1.67E-17 | 2.71E-01 | 5.94E-18 |
| 19 | 45298461 | rs10420519 | T | G | Intronic | *CBLC* | 0.0387 | 0.56 | 9.25E-10 | 3.43E-01 | 2.46E-10 |
| 19 | 45299199 | rs76560105 | T | G | Intronic | *CBLC* | 0.0360 | 0.56 | 1.50E-09 | 3.48E-01 | 4.39E-10 |
| 19 | 45302504 | rs148933445 | A | G | Intronic | *CBLC* | 0.0170 | 0.71 | 3.68E-08 | 1.51E-02 | 2.10E-07 |
| 19 | 45302778 | rs111794050 | G | A | Intronic | *CBLC* | 0.0449 | 0.50 | 9.36E-09 | 5.23E-01 | 4.14E-09 |
| 19 | 45309634 | rs112619823 | A | G | Intergenic | *CBLC,BCAM* | 0.0360 | 0.54 | 4.64E-09 | 4.63E-01 | 1.17E-09 |
| 19 | 45315445 | rs28399653 | A | G | Exonic (p.Arg77His) | *BCAM* | 0.0349 | 0.54 | 6.74E-09 | 6.13E-01 | 1.21E-09 |
| 19 | 45316588 | rs28399654 | A | G | Exonic (p.Val196Ile) | *BCAM* | 0.0338 | 0.54 | 5.73E-09 | 6.34E-01 | 1.06E-09 |
| 19 | 45318351 | rs28399657 | G | A | Intronic | *BCAM* | 0.0349 | 0.54 | 4.15E-09 | 6.06E-01 | 9.97E-10 |
| 19 | 45319631 | rs118147862 | A | G | Intronic | *BCAM* | 0.0378 | 0.91 | 3.29E-27 | 4.81E-01 | 5.90E-25 |
| 19 | 45345787 | rs111371860 | T | A | Intergenic | *BCAM,NECTIN2* | 0.0610 | 0.41 | 2.85E-09 | 6.01E-01 | 2.25E-08 |
| 19 | 45361646 | rs365653 | G | A | Intronic | *NECTIN2* | 0.1151 | 0.30 | 1.12E-08 | 3.13E-01 | 2.24E-06 |
| 19 | 45369203 | rs112422902 | A | G | Intronic | *NECTIN2* | 0.0342 | 0.81 | 1.92E-18 | 9.42E-01 | 1.92E-17 |
| 19 | 45382675 | rs41290120 | A | G | Intronic | *NECTIN2* | 0.0431 | 0.87 | 3.89E-28 | 8.00E-02 | 9.43E-26 |
| 19 | 45387034 | rs283808 | C | A | Intronic | *NECTIN2* | 0.0555 | 0.82 | 3.03E-25 | 3.20E-01 | 6.44E-23 |
| 19 | 45387459 | rs12972156 | G | C | Intronic | *NECTIN2* | 0.1282 | -0.29 | 1.43E-08 | 2.63E-05 | 3.95E-07 |
| 19 | 45388130 | rs34342646 | A | G | Intronic | *NECTIN2* | 0.1326 | -0.28 | 1.40E-08 | 2.45E-05 | 3.39E-07 |
| 19 | 45388241 | rs283810 | G | T | Intronic | *NECTIN2* | 0.0756 | 0.54 | 1.23E-15 | 8.87E-01 | 6.93E-15 |
| 19 | 45389174 | rs283813 | A | T | Intronic | *NECTIN2* | 0.0802 | 0.50 | 7.70E-15 | 9.05E-01 | 5.10E-14 |
| 19 | 45389596 | rs7254892 | A | G | Intronic | *NECTIN2* | 0.0398 | 1.07 | 1.33E-33 | 2.76E-01 | 1.61E-31 |
| 19 | 45392254 | rs6857 | T | C | UTR3 | *NECTIN2* | 0.1491 | -0.28 | 2.37E-09 | 5.58E-06 | 1.22E-07 |
| 19 | 45394336 | rs71352238 | C | T | Promoter | *TOMM40* | 0.1308 | -0.29 | 6.31E-09 | 1.02E-05 | 1.14E-07 |
| 19 | 45396144 | rs11556505 | T | C | Exonic (p.Phe131Leu) | *TOMM40* | 0.1292 | -0.30 | 6.06E-09 | 9.57E-06 | 1.08E-07 |
| 19 | 45397229 | rs1160983 | A | G | Exonic (p.Ser183Ser) | *TOMM40* | 0.0369 | 1.16 | 1.04E-36 | 2.56E-01 | 8.51E-35 |
| 19 | 45400747 | rs61679753 | A | T | Intronic | *TOMM40* | 0.0373 | 1.16 | 1.92E-37 | 2.19E-01 | 1.92E-35 |
| 19 | 45408628 | rs769446 | C | T | Promoter | *APOE* | 0.0919 | 0.49 | 8.33E-18 | 6.91E-03 | 5.59E-15 |
| 19 | 45408836 | rs405509 | T | G | Promoter | *APOE* | 0.4678 | -0.30 | 8.43E-19 | 4.23E-05 | 7.14E-17 |
| 19 | 45410002 | rs769449 | A | G | Intronic | *APOE* | 0.0983 | -0.34 | 1.64E-09 | 5.91E-07 | 2.26E-11 |
| 19 | 45411941 | rs429358 | C | T | Exonic (p.Cys112Arg) | *APOE* | 0.1254 | -0.35 | 8.73E-12 | 3.01E-08 | 1.53E-12 |
| 19 | 45412079 | rs7412 | T | C | Exonic (p.Arg158Cys) | *APOE* | 0.0800 | 1.12 | 4.74E-79 | NA | 8.06E-74 |
| 19 | 45413576 | rs75627662 | T | C | downstream | *APOE* | 0.1785 | 0.36 | 4.14E-17 | 1.55E-06 | 6.15E-15 |
| 19 | 45414399 | rs72654473 | A | C | Intergenic | *APOE,APOC1* | 0.1036 | 0.83 | 7.63E-54 | 1.83E-01 | 8.15E-52 |
| 19 | 45414451 | rs439401 | T | C | Intergenic | *APOE,APOC1* | 0.3732 | -0.29 | 1.47E-16 | 8.22E-06 | 1.61E-09 |
| 19 | 45415935 | rs7256200 | T | G | Intergenic | *APOE,APOC1* | 0.1030 | -0.35 | 4.17E-10 | 3.52E-07 | 3.25E-12 |
| 19 | 45416478 | rs584007 | A | G | Intergenic | *APOE,APOC1* | 0.3701 | -0.29 | 2.09E-16 | 7.78E-06 | 2.28E-09 |
| 19 | 45416741 | rs438811 | T | C | Promoter | *APOC1* | 0.2108 | 0.28 | 8.32E-12 | 1.69E-07 | 9.44E-10 |
| 19 | 45416831 | rs390082 | G | T | Promoter | *APOC1* | 0.1076 | 0.82 | 1.09E-52 | 1.60E-01 | 7.06E-51 |
| 19 | 45418790 | rs5117 | C | T | Intronic | *APOC1* | 0.2055 | 0.29 | 2.57E-12 | 5.88E-07 | 4.31E-10 |
| 19 | 45423944 | rs144311893 | T | C | Intergenic | *APOC1,APOC1P1* | 0.0163 | 1.40 | 8.66E-28 | 4.85E-04 | 2.31E-26 |
| 19 | 45425460 | rs157595 | A | G | Intergenic | *APOC1,APOC1P1* | 0.3929 | -0.22 | 1.76E-10 | 1.17E-08 | 2.12E-05 |
| 19 | 45426792 | rs141622900 | A | G | Intergenic | *APOC1,APOC1P1* | 0.0572 | 1.22 | 1.74E-68 | 2.01E-06 | 6.18E-65 |
| 19 | 45430280 | rs5112 | C | G | ncRNA Exonic | *APOC1P1* | 0.4615 | -0.34 | 2.36E-23 | 1.01E-20 | 3.79E-18 |
| 19 | 45431453 | rs114448690 | A | G | ncRNA_Intronic | *APOC1P1* | 0.0884 | 0.72 | 3.39E-34 | 1.53E-06 | 5.92E-29 |
| 19 | 45431636 | rs113345881 | G | A | ncRNA_Intronic | *APOC1P1* | 0.1021 | 0.59 | 1.32E-25 | 4.68E-04 | 2.79E-23 |
| 19 | 45431658 | rs8106813 | A | G | ncRNA_Intronic | *APOC1P1* | 0.4898 | -0.33 | 1.81E-22 | 6.23E-19 | 1.29E-16 |
| 19 | 45436657 | rs28795074 | A | G | Intergenic | *APOC1P1,APOC4* | 0.0844 | 0.73 | 2.57E-33 | 7.93E-06 | 3.98E-28 |
| 19 | 45439163 | rs35136575 | G | C | Intergenic | *APOC1P1,APOC4* | 0.2653 | -0.38 | 6.34E-24 | 1.09E-18 | NA |
| 19 | 45478352 | rs146390218 | G | A | Intronic | *CLPTM1* | 0.0214 | 0.68 | 2.61E-09 | 2.91E-01 | 1.12E-07 |
| 19 | 45491032 | rs117261169 | T | C | Intronic | *CLPTM1* | 0.0128 | 1.04 | 1.05E-12 | 8.24E-02 | 4.86E-11 |

**Supplementary Table S2:** List of 14 SNPs on chromosome 11 with suggestive GWS p-value including the top SNP (116648917) and then conditioned on the top SNP in this region. Red color indicates the four independent signals in this region based on LD.

| **CHR** | **Position (GRCh37)** | **GENE** | **SNP** | **Consequence** | **A1** | **MAF** | **β-value** | **SE** | **P-value** | **Conditioned on rs964184**  **β-value** | **Conditioned on rs964184 P-value** |
| --- | --- | --- | --- | --- | --- | --- | --- | --- | --- | --- | --- |
| 11 | 116596129 | *LINC02702,BUD13* | rs11216117 | Intergenic | A | 0.074 | 0.33 | 0.06575 | 7.68E-07 | 0.165 | 4.44E-02 |
| 11 | 116599900 | *LINC02702,BUD13* | rs61372904 | Intergenic | G | 0.073 | 0.33 | 0.06602 | 5.37E-07 | 0.171 | 3.91E-02 |
| 11 | 116600400 | *LINC02702,BUD13* | rs12279373 | Intergenic | G | 0.074 | 0.33 | 0.0659 | 7.34E-07 | 0.165 | 4.58E-02 |
| 11 | 116603724 | *LINC02702,BUD13* | rs12272004 | Intergenic | A | 0.073 | 0.33 | 0.06596 | 5.50E-07 | 0.170 | 4.07E-02 |
| 11 | 116623659 | *BUD13* | rs180327 | Intronic | C | 0.378 | 0.17 | 0.03424 | 3.86E-07 | 0.102 | 1.10E-02 |
| 11 | 116624703 | *BUD13* | rs180326 | Intronic | G | 0.372 | 0.18 | 0.03441 | 2.62E-07 | 0.105 | 9.98E-03 |
| 11 | 116635613 | *BUD13* | rs60972755 | Intronic | A | 0.068 | 0.34 | 0.06906 | 9.56E-07 | 0.155 | 8.59E-02 |
| 11 | 116648917 | *ZPR1/ZNF259* | rs964184 | UTR3 | G | 0.141 | 0.27 | 0.04801 | 2.58E-08 | NA | NA |
| 11 | 116649538 | *ZPR1/ZNF259* | rs61905116 | UTR3 | G | 0.062 | 0.36 | 0.07022 | 4.40E-07 | 0.178 | 5.03E-02 |
| 11 | 116655600 | *ZPR1/ZNF259* | rs35120633 | Exonic (p.A264V) | A | 0.061 | 0.36 | 0.07057 | 3.48E-07 | 0.187 | 3.97E-02 |
| 11 | 116658031 | *ZPR1ZNF259* | rs12285095 | Intronic | G | 0.066 | 0.34 | 0.06968 | 8.36E-07 | 0.161 | 7.64E-02 |
| 11 | 116662331 | *APOA5* | rs12287066 | Exonic (p.I44I) | T | 0.066 | 0.35 | 0.06985 | 6.45E-07 | 0.169 | 6.30E-02 |
| 11 | 116662407 | *APOA5* | rs3135506 | Exonic (p.S19W) | C | 0.062 | 0.36 | 0.07067 | 3.31E-07 | 0.189 | 3.70E-02 |
| 11 | 116893724 | *SIK3* | rs4936359 | Intronic | A | 0.127 | 0.25 | 0.04918 | 4.85E-07 | 0.186 | 2.83E-04 |

**Supplementary Table S3:** List of pre-defined 116 lead SNPs associated with plasma ApoE levels used in SNP2GENE

| **CHR** | **Position (GRCh37)** | **SNP** | **P** | **A1** | **MAF** | **BETA** | **Consequence** | **GENE** |
| --- | --- | --- | --- | --- | --- | --- | --- | --- |
| 1 | 29124928 | rs186224245 | 2.34E-09 | G | 0.01297 | 0.8739 | Intergenic | *YTHDF2,OPRD1* |
| 1 | 29146455 | rs114661586 | 5.36E-10 | A | 0.01261 | 0.921 | Intronic | *OPRD1* |
| 3 | 44452921 | rs113978340 | 3.78E-07 | A | 0.01224 | 0.7731 | Intergenic | *TCAIM,C3orf86* |
| 3 | 44706990 | rs75692653 | 9.16E-07 | A | 0.02174 | 0.5571 | ncRNA_Intronic | *ZKSCAN7-AS1* |
| 3 | 186335056 | rs140827890 | 7.55E-07 | A | 0.01023 | 0.8084 | Exonic (p.Ala164Thr) | *AHSG* |
| 4 | 23343005 | rs142344853 | 4.31E-08 | C | 0.01334 | 0.7963 | Intergenic | *GBA3,PPARGC1A* |
| 5 | 57673528 | rs147678095 | 7.88E-08 | A | 0.02265 | 0.5964 | Intergenic | *LINC02101,PLK2* |
| 5 | 57758618 | rs116255239 | 5.43E-08 | A | 0.02357 | 0.5972 | Intergenic | *PLK2,GAPT* |
| 5 | 57769068 | rs72758175 | 5.50E-08 | G | 0.02393 | 0.5924 | Intergenic | *PLK2,GAPT* |
| 6 | 63086756 | rs72872437 | 6.01E-07 | A | 0.01151 | 0.7882 | Intergenic | *KHDRBS2,LGSN* |
| 6 | 63167464 | rs151023032 | 5.70E-07 | A | 0.01261 | 0.7537 | Intergenic | *KHDRBS2,LGSN* |
| 7 | 11150689 | rs74425508 | 1.23E-07 | G | 0.02338 | 0.6131 | Intronic | *PHF14* |
| 7 | 11170450 | rs149497036 | 9.67E-09 | G | 0.01772 | 0.7216 | Intronic | *PHF14* |
| 7 | 118611279 | rs4470965 | 8.49E-07 | C | 0.02156 | 0.5984 | Intergenic | *ANKRD7,LINC02476* |
| 9 | 73033045 | rs559608259 | 3.20E-07 | C | 0.01224 | 0.7833 | Intergenic | *KLF9,TRPM3* |
| 9 | 73033812 | rs117989075 | 3.29E-07 | A | 0.01242 | 0.7767 | Intergenic | *KLF9,TRPM3* |
| 11 | 39787794 | rs71474365 | 3.06E-07 | T | 0.01078 | 0.844 | Intergenic | *LINC01493,LRRC4C* |
| 11 | 116596129 | rs11216117 | 7.68E-07 | A | 0.07399 | 0.3258 | Intergenic | *LINC02702,BUD13* |
| 11 | 116599900 | rs61372904 | 5.37E-07 | G | 0.07271 | 0.3318 | Intergenic | *LINC02702,BUD13* |
| 11 | 116600400 | rs12279373 | 7.34E-07 | G | 0.07362 | 0.3271 | Intergenic | *LINC02702,BUD13* |
| 11 | 116603724 | rs12272004 | 5.50E-07 | A | 0.07344 | 0.3312 | Intergenic | *LINC02702,BUD13* |
| 11 | 116623659 | rs180327 | 3.86E-07 | C | 0.3782 | 0.1742 | Intronic | *BUD13* |
| 11 | 116624703 | rs180326 | 2.62E-07 | G | 0.3721 | 0.1777 | Intronic | *BUD13* |
| 11 | 116635613 | rs60972755 | 9.56E-07 | A | 0.06796 | 0.3393 | Intronic | *BUD13* |
| 11 | 116648917 | rs964184 | 2.58E-08 | G | 0.1408 | 0.2681 | UTR3 | *ZPR1* |
| 11 | 116649538 | rs61905116 | 4.40E-07 | G | 0.06211 | 0.3556 | UTR3 | *ZPR1* |
| 11 | 116655600 | rs35120633 | 3.48E-07 | A | 0.06138 | 0.3605 | Exonic (p.Ala264Val) | *ZPR1* |
| 11 | 116658031 | rs12285095 | 8.36E-07 | G | 0.06558 | 0.3441 | Intronic | *ZPR1* |
| 11 | 116662331 | rs12287066 | 6.45E-07 | T | 0.06595 | 0.3485 | Exonic (p.Ile44 Ile) | *APOA5* |
| 11 | 116662407 | rs3135506 | 3.31E-07 | C | 0.06211 | 0.3617 | Exonic (p.Ser19Trp) | *APOA5* |
| 11 | 116893724 | rs4936359 | 4.85E-07 | A | 0.1268 | 0.2481 | Intronic | *SIK3* |
| 12 | 55415955 | rs2710701 | 2.21E-07 | G | 0.02265 | 0.6052 | Intronic | *NEUROD4* |
| 12 | 55416749 | rs2656806 | 1.55E-07 | G | 0.02558 | 0.605 | Intronic | *NEUROD4* |
| 12 | 55429513 | rs2633417 | 1.78E-07 | A | 0.02539 | 0.6083 | Intergenic | *NEUROD4,OR9K2* |
| 12 | 58530833 | rs2470341 | 4.64E-07 | C | 0.01078 | 1.731 | Intergenic | *LINC02403,LINC02388* |
| 12 | 58530967 | rs141952629 | 8.19E-07 | G | 0.01242 | 1.654 | Intergenic | *LINC02403,LINC02388* |
| 12 | 58531579 | rs2659650 | 8.19E-07 | G | 0.01261 | 1.654 | Intergenic | *LINC02403,LINC02388* |
| 12 | 58531728 | rs2653860 | 8.19E-07 | A | 0.01224 | 1.654 | Intergenic | *LINC02403,LINC02388* |
| 17 | 73582589 | rs144494783 | 2.01E-07 | T | 0.03416 | 0.476 | Intergenic | *LLGL2,MYO15B* |
| 19 | 45134682 | rs62119267 | 2.50E-07 | C | 0.01882 | 0.6247 | Intronic | *IGSF23* |
| 19 | 45169408 | rs62119319 | 3.27E-07 | T | 0.01955 | 0.6075 | UTR3 | *PVR* |
| 19 | 45197732 | rs62120566 | 5.60E-08 | G | 0.01918 | 0.6454 | Intergenic | *CEACAM19,CEACAM16* |
| 19 | 45198477 | rs10422568 | 5.46E-07 | T | 0.0411 | 0.4334 | Intergenic | *CEACAM19,CEACAM16* |
| 19 | 45200210 | rs75974038 | 4.37E-07 | T | 0.04037 | 0.4389 | Intergenic | *CEACAM19,CEACAM16* |
| 19 | 45231821 | rs1551891 | 3.41E-07 | A | 0.08403 | 0.3136 | Intergenic | *CEACAM16,BCL3* |
| 19 | 45232161 | rs62117160 | 1.03E-14 | A | 0.03507 | 0.6861 | Intergenic | *CEACAM16,BCL3* |
| 19 | 45233385 | rs62117161 | 1.83E-07 | G | 0.08422 | 0.3208 | Intergenic | *CEACAM16,BCL3* |
| 19 | 45239536 | rs62117162 | 1.02E-07 | A | 0.08604 | 0.3285 | Intergenic | *CEACAM16,BCL3* |
| 19 | 45242173 | rs1531517 | 1.47E-08 | A | 0.06777 | 0.3938 | Intergenic | *CEACAM16,BCL3* |
| 19 | 45242740 | rs2927439 | 1.28E-08 | G | 0.3412 | 0.2041 | Intergenic | *CEACAM16,BCL3* |
| 19 | 45242967 | rs62117204 | 2.31E-08 | T | 0.06467 | 0.3885 | Intergenic | *CEACAM16,BCL3* |
| 19 | 45247627 | rs4803750 | 3.29E-09 | G | 0.06485 | 0.4113 | Intergenic | *CEACAM16,BCL3* |
| 19 | 45249509 | rs115785198 | 4.12E-08 | T | 0.06321 | 0.3824 | Intergenic | *CEACAM16,BCL3* |
| 19 | 45255266 | rs62117205 | 5.86E-09 | C | 0.06394 | 0.4037 | Intronic | *BCL3* |
| 19 | 45295886 | rs113330691 | 1.32E-09 | A | 0.03727 | 0.5513 | Intronic | *CBLC* |
| 19 | 45296364 | rs112450640 | 1.67E-17 | A | 0.01863 | 1.056 | Intronic | *CBLC* |
| 19 | 45298461 | rs10420519 | 9.25E-10 | T | 0.03873 | 0.5591 | Intronic | *CBLC* |
| 19 | 45299199 | rs76560105 | 1.50E-09 | T | 0.03599 | 0.556 | Intronic | *CBLC* |
| 19 | 45302504 | rs148933445 | 3.68E-08 | A | 0.01699 | 0.7064 | Intronic | *CBLC* |
| 19 | 45302778 | rs111794050 | 9.36E-09 | G | 0.04494 | 0.5008 | Intronic | *CBLC* |
| 19 | 45303311 | rs10418198 | 7.87E-08 | A | 0.03745 | 0.4906 | Intronic | *CBLC* |
| 19 | 45309634 | rs112619823 | 4.64E-09 | A | 0.03599 | 0.5352 | Intergenic | *CBLC,BCAM* |
| 19 | 45315445 | rs28399653 | 6.74E-09 | A | 0.03489 | 0.5375 | Exonic (p.Arg77His) | *BCAM* |
| 19 | 45316588 | rs28399654 | 5.73E-09 | A | 0.0338 | 0.543 | Exonic (p.Val196Ile) | *BCAM* |
| 19 | 45318351 | rs28399657 | 4.15E-09 | G | 0.03489 | 0.5449 | Intronic | *BCAM* |
| 19 | 45319631 | rs118147862 | 3.29E-27 | A | 0.03782 | 0.914 | Intronic | *BCAM* |
| 19 | 45345787 | rs111371860 | 2.85E-09 | T | 0.06102 | 0.4093 | Intergenic | *BCAM,NECTIN2* |
| 19 | 45361646 | rs365653 | 1.12E-08 | G | 0.1151 | 0.2981 | Intronic | *NECTIN2* |
| 19 | 45369203 | rs112422902 | 1.92E-18 | A | 0.03416 | 0.8094 | Intronic | *NECTIN2* |
| 19 | 45382675 | rs41290120 | 3.89E-28 | A | 0.04311 | 0.8708 | Intronic | *NECTIN2* |
| 19 | 45387034 | rs283808 | 3.03E-25 | C | 0.05554 | 0.8162 | Intronic | *NECTIN2* |
| 19 | 45387459 | rs12972156 | 1.43E-08 | G | 0.1282 | -0.2881 | Intronic | *NECTIN2* |
| 19 | 45388130 | rs34342646 | 1.40E-08 | A | 0.1326 | -0.2834 | Intronic | *NECTIN2* |
| 19 | 45388241 | rs283810 | 1.23E-15 | G | 0.07563 | 0.5353 | Intronic | *NECTIN2* |
| 19 | 45389174 | rs283813 | 7.70E-15 | A | 0.0802 | 0.5049 | Intronic | *NECTIN2* |
| 19 | 45389596 | rs7254892 | 1.33E-33 | A | 0.03982 | 1.07 | Intronic | *NECTIN2* |
| 19 | 45392254 | rs6857 | 2.37E-09 | T | 0.1491 | -0.2845 | UTR3 | *NECTIN2* |
| 19 | 45394336 | rs71352238 | 6.31E-09 | C | 0.1308 | -0.2916 | Upstream | *TOMM40* |
| 19 | 45395844 | rs34095326 | 1.05E-07 | A | 0.1018 | -0.2956 | Intronic | *TOMM40* |
| 19 | 45396144 | rs11556505 | 6.06E-09 | T | 0.1292 | -0.2965 | Exonic (p.Phe131Phe) | *TOMM40* |
| 19 | 45397229 | rs1160983 | 1.04E-36 | A | 0.0369 | 1.158 | Exonic (p.Ser183Ser) | *TOMM40* |
| 19 | 45400747 | rs61679753 | 1.92E-37 | A | 0.03727 | 1.157 | Intronic | *TOMM40* |
| 19 | 45408628 | rs769446 | 8.33E-18 | C | 0.09189 | 0.4942 | Upstream | *APOE* |
| 19 | 45408836 | rs405509 | 8.43E-19 | T | 0.4678 | -0.2961 | Upstream | *APOE* |
| 19 | 45409167 | rs440446 | 2.60E-07 | C | 0.3617 | -0.1783 | Exonic (p.Asn14Lys) | *APOE* |
| 19 | 45410002 | rs769449 | 1.64E-09 | A | 0.09828 | -0.3443 | Intronic | *APOE* |
| 19 | 45411941 | rs429358 | 8.73E-12 | C | 0.1254 | -0.352 | Exonic (p.Cys112Arg) | *APOE* |
| 19 | 45412079 | rs7412 | 4.74E-79 | T | 0.08004 | 1.116 | Exonic (p. Arg158Cys) | *APOE* |
| 19 | 45413576 | rs75627662 | 4.14E-17 | T | 0.1785 | 0.3647 | Downstream | *APOE* |
| 19 | 45414399 | rs72654473 | 7.63E-54 | A | 0.1036 | 0.8289 | Intergenic | *APOE,APOC1* |
| 19 | 45414451 | rs439401 | 1.47E-16 | T | 0.3732 | -0.2862 | Intergenic | *APOE,APOC1* |
| 19 | 45415935 | rs7256200 | 4.17E-10 | T | 0.103 | -0.3522 | Intergenic | *APOE,APOC1* |
| 19 | 45416478 | rs584007 | 2.09E-16 | A | 0.3701 | -0.2857 | Intergenic | *APOE,APOC1* |
| 19 | 45416741 | rs438811 | 8.32E-12 | T | 0.2108 | 0.2817 | Upstream | *APOC1* |
| 19 | 45416831 | rs390082 | 1.09E-52 | G | 0.1076 | 0.8167 | Upstream | *APOC1* |
| 19 | 45418790 | rs5117 | 2.57E-12 | C | 0.2055 | 0.2884 | Intronic | *APOC1* |
| 19 | 45421254 | rs12721046 | 2.34E-07 | A | 0.1253 | -0.2641 | Intronic | *APOC1* |
| 19 | 45422160 | rs12721051 | 6.45E-08 | G | 0.1535 | -0.2535 | Intronic | *APOC1* |
| 19 | 45422946 | rs4420638 | 6.96E-08 | G | 0.1566 | -0.2528 | Downstream | *APOC1* |
| 19 | 45423944 | rs144311893 | 8.66E-28 | T | 0.01626 | 1.395 | Intergenic | *APOC1,APOC1P1* |
| 19 | 45425460 | rs157595 | 1.76E-10 | A | 0.3929 | -0.2203 | Intergenic | *APOC1,APOC1P1* |
| 19 | 45426792 | rs141622900 | 1.74E-68 | A | 0.05718 | 1.221 | Intergenic | *APOC1,APOC1P1* |
| 19 | 45427125 | rs111789331 | 2.09E-07 | A | 0.1264 | -0.2634 | Intergenic | *APOC1,APOC1P1* |
| 19 | 45428234 | rs66626994 | 1.40E-07 | A | 0.1286 | -0.2671 | Intergenic | *APOC1,APOC1P1* |
| 19 | 45430280 | rs5112 | 2.36E-23 | C | 0.4615 | -0.3367 | ncRNA_Exonic | *APOC1P1* |
| 19 | 45431453 | rs114448690 | 3.39E-34 | A | 0.08842 | 0.7209 | ncRNA_Intronic | *APOC1P1* |
| 19 | 45431636 | rs113345881 | 1.32E-25 | G | 0.1021 | 0.5913 | ncRNA_Intronic | *APOC1P1* |
| 19 | 45431658 | rs8106813 | 1.81E-22 | A | 0.4898 | -0.3296 | ncRNA_Intronic | *APOC1P1* |
| 19 | 45432505 | rs9636134 | 3.05E-07 | T | 0.375 | 0.1788 | ncRNA_Intronic | *APOC1P1* |
| 19 | 45436657 | rs28795074 | 2.57E-33 | A | 0.0844 | 0.7302 | Intergenic | *APOC1P1,APOC4* |
| 19 | 45439163 | rs35136575 | 6.34E-24 | G | 0.2653 | -0.3799 | Intergenic | *APOC1P1,APOC4* |
| 19 | 45478352 | rs146390218 | 2.61E-09 | G | 0.02137 | 0.6775 | Intronic | *CLPTM1* |
| 19 | 45491032 | rs117261169 | 1.05E-12 | T | 0.01279 | 1.035 | Intronic | *CLPTM1* |
| 20 | 6846108 | rs73894435 | 9.64E-09 | A | 0.01315 | 1.379 | Intergenic | *BMP2,LINC01428* |
| 20 | 6846987 | rs73894436 | 9.64E-09 | T | 0.01297 | 1.379 | Intergenic | *BMP2,LINC01428* |
| 20 | 6848770 | rs6054568 | 9.64E-09 | T | 0.0148 | 1.379 | Intergenic | *BMP2,LINC01428* |

**Supplementary Table S4:** List of 79 prioritized genes mapped by SNP2GENE

| **Gene ID** | **CHR** | **Start** | **End** | **Type** | **IndSigSNPs** |
| --- | --- | --- | --- | --- | --- |
| *OPRD1* | 1 | 29138654 | 29190208 | protein_coding | rs186224245;rs114661586 |
| *ZNF197* | 3 | 44626380 | 44689963 | protein_coding | rs113978340 |
| *KIF15* | 3 | 44803209 | 44914868 | protein_coding | rs75692653 |
| *AHSG* | 3 | 186330712 | 186339107 | protein_coding | rs140827890 |
| *ZKSCAN7* | 3 | 44596685 | 44624975 | protein_coding | rs113978340 |
| *TCAIM* | 3 | 44379611 | 44450943 | protein_coding | rs113978340 |
| *FETUB* | 3 | 186353758 | 186370930 | protein_coding | rs140827890 |
| *TBCCD1* | 3 | 186263862 | 186288332 | protein_coding | rs140827890 |
| *KIAA1143* | 3 | 44779153 | 44803154 | protein_coding | rs75692653 |
| *ZNF35* | 3 | 44690219 | 44702283 | protein_coding | rs75692653 |
| *DNAJB11* | 3 | 186285192 | 186315061 | protein_coding | rs140827890 |
| *PLK2* | 5 | 57749809 | 57756087 | protein_coding | rs147678095;rs116255239;rs72758175 |
| *KHDRBS2* | 6 | 62389865 | 62996132 | protein_coding | rs151023032 |
| *PHF14* | 7 | 11013499 | 11209250 | protein_coding | rs74425508;rs149497036 |
| *MAMDC2* | 9 | 72658497 | 72841886 | protein_coding | rs559608259;rs117989075 |
| *KLF9* | 9 | 72999503 | 73029540 | protein_coding | rs559608259;rs117989075 |
| *CEP164* | 11 | 117185273 | 117283984 | protein_coding | rs12279373 |
| *APOA5* | 11 | 116660083 | 116663136 | protein_coding | rs11216117;rs61372904;rs12279373;rs12272004;rs60972755;rs61905116;rs35120633;rs12285095;rs12287066;rs3135506;rs964184 |
| *APOA4* | 11 | 116691419 | 116694022 | protein_coding | rs12279373;rs4936359 |
| *APOC3* | 11 | 116700422 | 116703788 | protein_coding | rs12279373;rs4936359 |
| *BUD13* | 11 | 116618886 | 116643704 | protein_coding | rs11216117;rs61372904;rs12279373;rs12272004;rs60972755;rs61905116;rs35120633;rs12285095;rs12287066;rs3135506;rs180327;rs180326;rs964184 |
| *ZNF259* | 11 | 116648436 | 116658766 | protein_coding | rs11216117;rs61372904;rs12279373;rs12272004;rs60972755;rs61905116;rs35120633;rs12285095;rs12287066;rs3135506;rs964184 |
| *APOA1* | 11 | 116706467 | 116708666 | protein_coding | rs4936359 |
| *SIK3* | 11 | 116714118 | 116969153 | protein_coding | rs4936359 |
| *DSCAML1* | 11 | 117298489 | 117688240 | protein_coding | rs12279373 |
| *MARS* | 12 | 57869228 | 57911352 | protein_coding | rs2470341;rs141952629;rs2659650;rs2653860 |
| *AVIL* | 12 | 58191159 | 58212487 | protein_coding | rs2470341;rs141952629;rs2659650;rs2653860 |
| *SLC26A10* | 12 | 58013310 | 58019934 | protein_coding | rs2470341;rs141952629;rs2659650;rs2653860 |
| *ARHGAP9* | 12 | 57866038 | 57882597 | protein_coding | rs2470341;rs141952629;rs2659650;rs2653860 |
| *TSFM* | 12 | 58176372 | 58201854 | protein_coding | rs2470341;rs141952629;rs2659650;rs2653860 |
| *OS9* | 12 | 58087738 | 58115340 | protein_coding | rs2470341;rs141952629;rs2659650;rs2653860 |
| *GLI1* | 12 | 57853918 | 57866045 | protein_coding | rs2470341;rs141952629;rs2659650;rs2653860 |
| *CYP27B1* | 12 | 58156117 | 58162769 | protein_coding | rs2470341;rs141952629;rs2659650;rs2653860 |
| *ARHGEF25* | 12 | 58003963 | 58013162 | protein_coding | rs2470341;rs141952629;rs2659650;rs2653860 |
| *INHBE* | 12 | 57846106 | 57853063 | protein_coding | rs2470341;rs141952629;rs2659650;rs2653860 |
| *XRCC6BP1* | 12 | 58335324 | 58351052 | protein_coding | rs2470341;rs141952629;rs2659650;rs2653860 |
| *STAC3* | 12 | 57637236 | 57644976 | protein_coding | rs2470341;rs141952629;rs2659650;rs2653860 |
| *METTL21B* | 12 | 58165275 | 58176324 | protein_coding | rs2470341;rs141952629;rs2659650;rs2653860 |
| *METTL1* | 12 | 58162254 | 58166576 | protein_coding | rs2470341;rs141952629;rs2659650;rs2653860 |
| *SHMT2* | 12 | 57623110 | 57628718 | protein_coding | rs2470341;rs141952629;rs2659650;rs2653860 |
| *PIP4K2C* | 12 | 57984957 | 57997198 | protein_coding | rs2470341;rs141952629;rs2659650;rs2653860 |
| *NDUFA4L2* | 12 | 57628686 | 57634498 | protein_coding | rs2470341;rs141952629;rs2659650;rs2653860 |
| *AGAP2-AS1* | 12 | 58120054 | 58122139 | protein_coding | rs2470341;rs141952629;rs2659650;rs2653860 |
| *B4GALNT1* | 12 | 58017193 | 58027138 | protein_coding | rs2470341;rs141952629;rs2659650;rs2653860 |
| *INHBC* | 12 | 57828543 | 57844611 | protein_coding | rs2470341;rs141952629;rs2659650;rs2653860 |
| *NEUROD4* | 12 | 55413729 | 55423798 | protein_coding | rs2710701;rs2656806;rs2633417 |
| *TSPAN31* | 12 | 58131796 | 58143994 | protein_coding | rs2470341;rs141952629;rs2659650;rs2653860 |
| *9-Mar* | 12 | 58148881 | 58154190 | protein_coding | rs2470341;rs141952629;rs2659650;rs2653860 |
| *CTDSP2* | 12 | 58213710 | 58240522 | protein_coding | rs2470341;rs141952629;rs2659650;rs2653860 |
| *LRIG3* | 12 | 59265931 | 59314303 | protein_coding | rs2470341;rs141952629;rs2659650;rs2653860 |
| *DDIT3* | 12 | 57910371 | 57914300 | protein_coding | rs2470341;rs141952629;rs2659650;rs2653860 |
| *DTX3* | 12 | 57998405 | 58003587 | protein_coding | rs2470341;rs141952629;rs2659650;rs2653860 |
| *CDK4* | 12 | 58141510 | 58149796 | protein_coding | rs2470341;rs141952629;rs2659650;rs2653860 |
| *MBD6* | 12 | 57914493 | 57923931 | protein_coding | rs2470341;rs141952629;rs2659650;rs2653860 |
| *R3HDM2* | 12 | 57643392 | 57824788 | protein_coding | rs2470341;rs141952629;rs2659650;rs2653860 |
| *DCTN2* | 12 | 57923885 | 57941114 | protein_coding | rs2470341;rs141952629;rs2659650;rs2653860 |
| *AGAP2* | 12 | 58118980 | 58135940 | protein_coding | rs2470341;rs141952629;rs2659650;rs2653860 |
| *KIF5A* | 12 | 57943781 | 57980415 | protein_coding | rs2470341;rs141952629;rs2659650;rs2653860 |
| *RP11-123K3.4* | 12 | 57643392 | 57690267 | protein_coding | rs2470341;rs141952629;rs2659650;rs2653860 |
| *AC126614.1* | 12 | 57810198 | 57810536 | protein_coding | rs2470341;rs141952629;rs2659650;rs2653860 |
| *RP11-571M6.15* | 12 | 58166811 | 58180829 | protein_coding | rs2470341;rs141952629;rs2659650;rs2653860 |
| *RP11-362K2.2* | 12 | 58937907 | 59206842 | protein_coding | rs2470341;rs141952629;rs2659650;rs2653860 |
| *MYO15B* | 17 | 73584139 | 73622929 | protein_coding | rs144494783 |
| *PVR* | 19 | 45147098 | 45166850 | protein_coding | rs62119267;rs62119319;rs62120566;rs10422568;rs75974038 |
| *CEACAM19* | 19 | 45165545 | 45187631 | protein_coding | rs62119267;rs62119319;rs62120566;rs10422568;rs75974038 |
| *APOC4* | 19 | 45445495 | 45452820 | protein_coding | rs8106813;rs114448690;rs113345881;rs28795074;rs35136575 |
| *CBLC* | 19 | 45281126 | 45303891 | protein_coding | rs113330691;rs112450640;rs10420519;rs76560105;rs111794050;rs10418198;rs112619823;rs28399653;rs28399654;rs28399657;rs148933445 |
| *CEACAM16* | 19 | 45202421 | 45213986 | protein_coding | rs10422568;rs75974038;rs62119267;rs62119319;rs62120566;rs1551891;rs62117161;rs62117162 |
| *APOE* | 19 | 45409011 | 45412650 | protein_coding | rs112422902;rs283808;rs7254892;rs1160983;rs61679753;rs405509;rs769446;rs440446;rs71352238;rs11556505;rs769449;rs429358;rs75627662;rs7256200;rs12721046;rs111789331;rs66626994;rs6857;rs438811;rs5117;rs12721051;rs4420638;rs7412;rs72654473;rs390082;rs141622900;rs439401;rs584007;rs157595 |
| *BCAM* | 19 | 45312328 | 45324673 | protein_coding | rs148933445;rs113330691;rs10420519;rs76560105;rs111794050;rs10418198;rs112619823;rs28399653;rs28399654;rs28399657;rs62117160;rs118147862;rs41290120 |
| *APOC1* | 19 | 45417504 | 45422606 | protein_coding | rs405509;rs769446;rs440446;rs71352238;rs11556505;rs769449;rs429358;rs75627662;rs7256200;rs12721046;rs111789331;rs66626994;rs6857;rs438811;rs5117;rs12721051;rs4420638;rs7412;rs72654473;rs390082;rs141622900;rs439401;rs584007;rs157595;rs144311893;rs5112;rs8106813;rs114448690;rs113345881;rs28795074 |
| *PVRL2* | 19 | 45349432 | 45392485 | protein_coding | rs111371860;rs365653;rs112422902;rs7254892;rs1160983;rs61679753;rs118147862;rs41290120;rs12972156;rs34342646;rs6857;rs71352238;rs34095326;rs11556505;rs283808;rs283810;rs283813;rs429358;rs769449;rs7256200;rs405509 |
| *BCL3* | 19 | 45250962 | 45263301 | protein_coding | rs1551891;rs62117161;rs62117162;rs1531517;rs62117204;rs4803750;rs115785198;rs62117205;rs2927439;rs148933445 |
| *TOMM40* | 19 | 45393826 | 45406946 | protein_coding | rs12972156;rs34342646;rs6857;rs71352238;rs34095326;rs11556505;rs283808;rs7254892;rs1160983;rs61679753;rs283810;rs283813;rs112422902;rs429358;rs769449;rs7256200;rs405509;rs769446;rs440446;rs75627662;rs12721046;rs111789331;rs66626994;rs438811;rs5117;rs12721051;rs4420638;rs7412;rs72654473;rs390082;rs141622900;rs439401;rs584007;rs157595 |
| *CLPTM1* | 19 | 45457842 | 45496599 | protein_coding | rs146390218;rs117261169 |
| *MARK4* | 19 | 45582546 | 45808541 | protein_coding | rs117261169 |
| *APOC4-APOC2* | 19 | 45445495 | 45452822 | protein_coding | rs8106813;rs114448690;rs113345881;rs28795074;rs35136575 |
| *IGSF23* | 19 | 45116940 | 45140081 | protein_coding | rs62119267;rs62119319;rs62120566 |
| *PPP1R37* | 19 | 45594654 | 45651335 | protein_coding | rs117261169 |

**Supplementary Table S5:** GWAS Catalog reported genes from FUMA-GWAS

| **Gene Set** | **N** | **n** | **P-value** | **adjusted P** | **Genes** |
| --- | --- | --- | --- | --- | --- |
| Alzheimer's disease or HDL levels (pleiotropy) | 53 | 16 | 9.34e-27 | 1.69e-23 | *IGSF23, PVR, CEACAM19, CEACAM16, BCL3, CBLC, BCAM, PVRL2, TOMM40, APOE, APOC1, APOC4-APOC2, APOC4, CLPTM1, MARK4, PPP1R37* |
| Body mass index x age interaction | 37 | 12 | 1.06e-20 | 9.59e-18 | *IGSF23, PVR, BCL3, CBLC, BCAM, PVRL2, TOMM40, APOE, APOC1, CLPTM1, MARK4, PPP1R37* |
| Body mass index (age>50) | 59 | 12 | 5.96e-18 | 3.22e-15 | *IGSF23, PVR, BCL3, CBLC, BCAM, PVRL2, TOMM40, APOE, APOC1, CLPTM1, MARK4, PPP1R37* |
| Cerebrospinal AB1-42 levels in Alzheimer's disease dementia | 18 | 9 | 7.09e-18 | 3.22e-15 | *CBLC, BCAM, PVRL2, TOMM40, APOE, APOC1, APOC4-APOC2, APOC4, CLPTM1* |
| Hippocampal volume in Alzheimer's disease dementia* | 52 | 11 | 9.64e-17 | 3.50e-14 | *CEP164, DSCAML1, ARHGAP9, BCAM, PVRL2, TOMM40, APOE, APOC1, APOC4-APOC2, APOC4, CLPTM1* |
| Cerebrospinal fluid t-tau levels in mild cognitive impairment | 16 | 8 | 5.30e-16 | 1.37e-13 | *BCAM, PVRL2, TOMM40, APOE, APOC1, APOC4-APOC2, APOC4, CLPTM1* |
| Cerebrospinal AB1-42 levels in mild cognitive impairment | 16 | 8 | 5.30e-16 | 1.37e-13 | *BCAM, PVRL2, TOMM40, APOE, APOC1, APOC4-APOC2, APOC4, CLPTM1* |
| Body mass index x sex x age interaction (4df test) | 91 | 12 | 1.52e-15 | 3.45e-13 | *IGSF23, PVR, BCL3, CBLC, BCAM, PVRL2, TOMM40, APOE, APOC1, CLPTM1, MARK4, PPP1R37* |
| Alzheimer's disease or family history of Alzheimer's disease | 48 | 10 | 3.05e-15 | 5.60e-13 | *IGSF23, PVR, BCL3, CBLC, BCAM, PVRL2, TOMM40, APOE, CLPTM1, MARK4* |
| Logical memory (immediate recall) | 19 | 8 | 3.08e-15 | 5.60e-13 | *BCAM, PVRL2, TOMM40, APOE, APOC1, APOC4-APOC2, APOC4, CLPTM1* |
| Logical memory (delayed recall) | 21 | 8 | 8.25e-15 | 1.36e-12 | *BCAM, PVRL2, TOMM40, APOE, APOC1, APOC4-APOC2, APOC4, CLPTM1* |
| Cerebrospinal fluid t-tau levels | 23 | 8 | 1.97e-14 | 2.99e-12 | *BCAM, PVRL2, TOMM40, APOE, APOC1, APOC4-APOC2, APOC4, CLPTM1* |
| Lipid traits | 24 | 8 | 2.95e-14 | 4.12e-12 | *BUD13, APOA5, APOA4, APOC3, APOA1, TOMM40, APOE, APOC1* |
| Cerebrospinal fluid AB1-42 levels | 50 | 9 | 3.30e-13 | 4.28e-11 | *BCAM, PVRL2, TOMM40, APOE, APOC1, APOC4-APOC2, APOC4, CLPTM1, PPP1R37* |
| Cerebrospinal fluid p-tau levels in mild cognitive impairment | 21 | 7 | 1.30e-12 | 1.58e-10 | *BCAM, PVRL2, TOMM40, APOE, APOC1, APOC4, CLPTM1* |
| Triglycerides | 174 | 12 | 4.07e-12 | 4.61e-10 | *BUD13, ZNF259, APOA5, APOA4, APOC3, APOA1, SIK3, DSCAML1, R3HDM2, TOMM40, APOE, APOC1* |
| LDL cholesterol | 178 | 12 | 5.32e-12 | 5.68e-10 | *BUD13, ZNF259, APOA5, APOA4, APOC3, APOA1, CBLC, BCAM, TOMM40, APOE, APOC1, APOC4* |
| Cerebrospinal fluid p-tau levels | 17 | 6 | 3.83e-11 | 3.86e-9 | *BCAM, PVRL2, APOE, APOC1, APOC4, CLPTM1* |
| Alzheimer's disease in hypertension | 9 | 5 | 1.07e-10 | 1.01e-8 | *BCAM, PVRL2, TOMM40, APOE, APOC1* |
| Triglyceride levels | 132 | 10 | 1.12e-10 | 1.01e-8 | *BUD13, ZNF259, APOA5, APOA4, APOC3, APOA1, SIK3, DSCAML1, APOE, APOC1* |
| LDL cholesterol levels | 95 | 9 | 1.34e-10 | 1.16e-8 | *APOA5, APOA4, APOC3, APOA1, CEACAM16, BCL3, TOMM40, APOE, APOC1* |
| Alzheimer's disease in hypertension-negative individuals | 10 | 5 | 2.14e-10 | 1.77e-8 | *BCAM, PVRL2, TOMM40, APOE, APOC1* |
| Delirium** | 44 | 7 | 3.99e-10 | 3.15e-8 | *BUD13, ZNF259, APOA5, APOA4, APOC3, APOA1, SIK3* |
| Hippocampal volume | 47 | 7 | 6.49e-10 | 4.91e-8 | *BCAM, PVRL2, TOMM40, APOE, APOC1, APOC4, CLPTM1* |
| Metabolic syndrome | 52 | 7 | 1.36e-9 | 9.87e-8 | *BUD13, ZNF259, APOA5, APOA4, SIK3, TOMM40, APOE* |

* *CEP164* and *DSCAML1* are in novel locus on chromosome 11; *ARHGAP9* is in novel locus on chromosome 12

** All red highlighted genes are in novel locus on chromosome 11

**Supplementary Table S6:** Significant association of reported 8 independent genome-wide significant AD-associated SNPs in the *APOE* region (*Nat Genet* 2019; 51:404-413) with plasma ApoE level in the current study.

|  |  |  |  |  | **AD case-control** | | **Plasma ApoE level** | |  |  |
| --- | --- | --- | --- | --- | --- | --- | --- | --- | --- | --- |
| **CHR** | **Position (GRCh37)** | **SNP** | **A1** | **MAF** | **β-value** | **P-value** | **β-value** | **P-value** | **Consequence** | **GENE** |
| 19 | 45251156 | rs2965169 | C | 0.409 | -0.035 | 1.04E-05 | 0.1394 | 5.56E-05 | Upstream | *BCL3* |
| 19 | 45324138 | rs28399637 | A | 0.294 | 0.061 | 2.25E-57 | -0.1493 | 3.99E-05 | Intronic | *BCAM* |
| 19 | 45341948 | rs10407439 | A | 0.295 | -0.038 | 1.71E-16 | 0.0945 | 0.01054 | Intergenic | *BCAM,NECTIN2* |
| 19 | 45351516 | rs41289512 | G | 0.0334 | 0.206 | 5.39E-276 | -0.424 | 5.35E-06 | Intronic | *NECTIN2* |
| 19 | 45363820 | rs138607350 | G | 0.0064 | 0.225 | 8.31E-59 | -0.3479 | 0.1013 | Intronic | *NECTIN2* |
| 19 | 45382034 | rs6859 | A | 0.4121 | 0.058 | 1.39E-48 | -0.1497 | 1.60E-05 | UTR3 | *NECTIN2* |
| 19 | 45384931 | rs79701229 | A | 0.0117 | 0.195 | 1.40E-53 | -0.1537 | 0.3172 | Intronic | *NECTIN2* |
| 19 | 45412955 | rs1081105 | C | 0.0232 | 0.220 | 2.94E-247 | -0.2431 | 0.0307 | Downstream | *APOE* |

**Supplementary Table S7:** Regional association of top AD loci (Neurotherapeutics 2022;19:152–172) with plasma ApoE level

| **Chr** | **Start BP** | **End BP** | **Locus** | **Total SNPs in the AD region** | **No. of SNPs P<0.05** | **Top AD-associated SNP in the region** | **Position BP** | **A1** | **A2** | **BETA** | **ApoE level**  **P-value** |
| --- | --- | --- | --- | --- | --- | --- | --- | --- | --- | --- | --- |
| 19 | 841398 | 1275987 | *ABCA7* | 1338 | 102 | *MED16*/rs117463628 | 874397 | A | G | 0.3618 | 9.88E-05 |
| 6 | 32261252 | 32826450 | *HLA/DRB5/DRB1* | 3235 | 390 | *TAP1*/rs17213812 | 32815516 | T | C | 0.2722 | 1.22E-04 |
| 14 | 92791068 | 93153606 | *SLC24A4* | 990 | 46 | *RIN3*/rs72631620 | 93049411 | A | G | 0.2813 | 2.02E-04 |
| 14 | 53109716 | 53417538 | *FERMT2* | 325 | 9 | *GNPNAT1,FERMT2*/rs62003490 | 53288310 | A | G | 0.1166 | 7.23E-04 |
| 7 | 143014244 | 143218545 | *EPHA1* | 385 | 16 | *EPHA1-AS1*/rs4621713 | 143131256 | C | A | -0.3296 | 7.94E-04 |
| 1 | 207669924 | 207814835 | *CR1* | 113 | 9 | *CR1*/rs115632000 | 207693902 | G | T | -0.2895 | 8.85E-04 |
| 3 | 154741991 | 154901493 | *MME* | 230 | 16 | *MME*/rs12490001 | 154809684 | G | A | 0.1955 | 1.56E-03 |
| 10 | 61786056 | 62493286 | *ANK3* | 1092 | 80 | *ANK3*/rs117193770 | 62248749 | A | C | 0.4622 | 2.04 E-03 |
| 11 | 59807796 | 60108278 | *MS4A6A* | 400 | 58 | *MS4A2,MS4A6A*/rs580064 | 59869119 | C | T | 0.1017 | 2.28E-03 |
| 2 | 106361369 | 106510728 | *NCK2* | 237 | 8 | *NCK2*/rs147765848 | 106370368 | C | T | 0.3913 | 2.49E-03 |
| 8 | 27094615 | 27402132 | *PTK2B* | 572 | 21 | *STMN4,TRIM35*/rs73239493 | 27121839 | A | G | -0.1984 | 3.59E-03 |
| 11 | 47186424 | 47868853 | *CELF1* | 611 | 14 | *MYBPC3*/rs11570057 | 47369760 | G | C | 0.2936 | 4.18E-03 |
| 2 | 233743532 | 234115739 | *INPP5D* | 787 | 53 | *INPP5D*/rs114306516 | 233949973 | A | G | 0.4186 | 4.44E-03 |
| 7 | 37780799 | 37939840 | *NME8* | 319 | 19 | *NME8*/rs79864825 | 37895267 | C | T | 0.2082 | 5.02E-03 |
| 8 | 27183710 | 27695123 | *CLU* | 820 | 26 | *ESCO2*/rs34929165 | 27636518 | T | C | -0.2935 | 7.50E-03 |
| 11 | 121328954 | 121502894 | *SORL1* | 266 | 2 | *SORL1*/rs77510550 | 121481196 | A | G | 0.1449 | 9.47E-03 |
| 11 | 85566157 | 85779310 | *PICALM* | 323 | 10 | *PICALM*/rs147642175 | 85763245 | T | G | 0.3872 | 1.36E-02 |
| 6 | 47445789 | 47688606 | *CD2AP* | 211 | 3 | *CD2AP,ADGRF2*/rs150665848 | 47605439 | A | G | -0.4125 | 1.36E-02 |
| 11 | 47376411 | 47400098 | *SPl1* | 42 | 1 | *SPI1*/rs142817897 | 47395672 | T | C | 0.3678 | 2.22E-02 |
| 20 | 54968038 | 55111371 | *CASS4* | 237 | 5 | *CSTF1*/rs2426620 | 54974993 | C | T | 0.3754 | 3.23E-02 |
| 2 | 127806605 | 127864546 | *BIN1* | 158 | 2 | *BIN1*/rs61748155 | 127809840 | A | C | -0.2445 | 4.29E-02 |

**Supplementary Table S8:** Association of common and low-frequency variants within 17 AD-associated genes implicated by rare variants in non-*APOE* regions with plasma ApoE levels.

| **CHR** | **Position (GRCh37)** | **SNP** | **A1** | **MAF** | **β-value** | **SE** | **P-value** | **LOC** | **GENE** |
| --- | --- | --- | --- | --- | --- | --- | --- | --- | --- |
| 1 | 956327 | rs111893801 | T | 0.064 | -0.149 | 0.068 | 2.87E-02 | Intronic | *AGRN* |
| 1 | 109851126 | rs413582 | T | 0.465 | -0.091 | 0.034 | 7.10E-03 | Intergenic | *MYBPHL,SORT1* |
| 1 | 109856843 | rs661278 | T | 0.057 | 0.197 | 0.073 | 7.17E-03 | Intronic | *SORT1* |
| 2 | 106322027 | rs72823359 | A | 0.026 | 0.265 | 0.107 | 1.31E-02 | Intergenic | *LOC285000,NCK2* |
| 2 | 106370368 | rs147765848 | C | 0.017 | 0.391 | 0.129 | 2.49E-03 | Intronic | *NCK2* |
| 2 | 106655249 | rs4851911 | G | 0.153 | 0.141 | 0.046 | 2.45E-03 | Intergenic | *NCK2,ECRG4* |
| 4 | 77138460 | rs920608 | C | 0.011 | 0.536 | 0.180 | 2.96E-03 | Intronic | *SCARB2/FAM47E* |
| 6 | 41138049 | rs12202176 | G | 0.018 | 0.257 | 0.127 | 4.27E-02 | Intergenic | *TREM2,TREML2* |
| 7 | 145744347 | rs1917649 | A | 0.171 | -0.150 | 0.044 | 7.42E-04 | Intergenic | *TPK1,CNTNAP2* |
| 7 | 146785080 | rs9640234 | A | 0.341 | -0.091 | 0.036 | 1.20E-02 | ncRNA_  intronic | *CNTNAP2-AS1* |
| 7 | 147319218 | rs75510933 | T | 0.01 | 0.897 | 0.274 | 1.08E-03 | Intronic | *CNTNAP2* |
| 7 | 148263038 | rs60750997 | T | 0.044 | -0.205 | 0.082 | 1.22E-02 | Intergenic | *CNTNAP2,C7orf33* |
| 16 | 81962833 | rs114484047 | C | 0.01 | 0.557 | 0.188 | 3.11E-03 | Intronic | *PLCG2* |
| 16 | 82002848 | rs12932876 | C | 0.064 | 0.152 | 0.070 | 3.09E-02 | Intergenic | *PLCG2,SDR42E1* |
| 16 | 86454210 | rs16941239 | A | 0.029 | 0.266 | 0.109 | 1.53E-02 | Intergenic | *FOXF1* |
| 17 | 61538148 | rs138190086 | A | 0.018 | -0.276 | 0.127 | 2.95E-02 | Intergenic | *ACE/CYB561* |
| 19 | 3405592 | rs9749589 | A | 0.153 | -0.099 | 0.047 | 3.39E-02 | Intronic | *NFIC* |

**Supplementary Table 9:** Association of novel plasma ApoE-associated SNPs in the *APOE* and non-*APOE* regions in this study with AD risk and amyloid-PET.

|  |  |  | **Plasma ApoE level** | | | **IGAP discovery AD**  **case-control^1^** | | | **Amyloid-PET^2^** | | |
| --- | --- | --- | --- | --- | --- | --- | --- | --- | --- | --- | --- |
| **CHR** | **Position GRCh37** | **SNP** | **A1** | **β-value** | **P** | **A1** | **β-value** | **P** | **A1** | **BETA** | **P** |
| 1 | 29146455 | rs114661586 | A | 0.92 | 5.40E-10 | A | 0.0658 | 0.2748 | NP |  |  |
| 4 | 23343005 | rs142344853 | C | 0.8 | 4.30E-08 | C | -0.0605 | 0.3886 | NP |  |  |
| 5 | 57769068 | rs72758175 | G | 0.624 | 5.50E-08 | G | 0.0134 | 0.7798 | NP |  |  |
| 7 | 11170450 | rs149497036 | G | 0.72 | 9.70E-09 | G | -0.0225 | 0.8456 | NP |  |  |
| 11 | 116648917 | rs964184 | G | 0.27 | 2.60E-08 | G | 0.0053 | 0.7973 | G | 0.014 | 4.30E-01 |
| 12 | 58530833 | rs2470341 | C | 1.73 | 4.60E-07 | NP |  |  | NP |  |  |
| 19 | 45431453 | rs114448690 | A | 0.72 | 3.40E-34 | A | -0.0134 | 0.7167 | NP |  |  |
| 19 | 45396144 | rs11556505 | T | -0.3 | 6.10E-09 | T | 0.9576 | 6.15E-575 | T | 0.128 | 2.03E-15 |
| 19 | 45423944 | rs144311893 | T | 1.4 | 8.70E-28 | T | -0.6358 | 7.60E-23 | NP |  |  |
| 19 | 45302504 | rs148933445 | A | 0.71 | 3.70E-08 | A | -0.5753 | 4.30E-19 | NP |  |  |
| 19 | 45425460 | rs157595 | A | -0.22 | 1.80E-10 | A | -0.4329 | 2.00E-143 | NP |  |  |
| 19 | 45414451 | rs439401 | T | -0.29 | 1.47E-16 | T | -0.3824 | 7.61E-128 | T | -0.0525 | 2.92E-05 |
| 19 | 45439163 | rs35136575 | G | -0.38 | 6.30E-24 | G | 0.0368 | 0.1697 | NP |  |  |
| 19 | 45408836 | rs405509 | T | -0.3 | 8.40E-19 | T | 0.3331 | 3.00E-114 | T | -0.021 | 5.82E-04 |
| 19 | 45411941 | rs429358 | C | -0.35 | 2.20E-11 | C | 1.2017 | 1.17E-881 | C | 0.181 | 9.09E-30 |
| 19 | 45430280 | rs5112 | C | -0.34 | 2.40E-23 | C | 0.0749 | 1.83E-04 | NP |  |  |
| 19 | 45412079 | rs7412 | T | 1.11 | 4.70E-79 | T | -0.4673 | 6.40E-53 | T | -0.09 | 6.57E-05 |
| 19 | 45408628 | rs769446 | C | 0.49 | 8.30E-18 | C | -0.2077 | 2.10E-09 | NP |  |  |
| 20 | 6846108 | rs73894435 | A | 1.37 | 9.60E-09 | A | -0.0398 | 0.7633 | NP |  |  |

NP= SNP does not present in the study

^1^ From *Nat Genet* 2019;51:414-430. We used data from the AD case-control IGAP discovery sample (20,344 cases and 52,427 controls); ^2^ From *Mol Psychiatry* 2021; 26:309-321.
